# Working With School‐Aged Children With Neurodisability and Oropharyngeal Dysphagia Who Require Mealtime Assistance: A Survey of Speech and Language Therapists’ Clinical Practice

**DOI:** 10.1111/1460-6984.70254

**Published:** 2026-04-29

**Authors:** Sally Morgan, Kathleen Mulligan, Kelly A Weir, Katerina Hilari

**Affiliations:** ^1^ School of Health & Medical Sciences City St George's, University of London London UK; ^2^ East London NHS Foundation Trust London UK; ^3^ Department of Audiology and Speech Pathology The University of Melbourne Carlton Victoria Australia; ^4^ Royal Children's Hospital Parkville Victoria Australia

**Keywords:** feeding disorder, paediatric feeding disorder, speech language pathology, survey, swallowing disorder

## Abstract

**Background:**

School‐aged children with neurodisability and oropharyngeal dysphagia who need mealtime assistance have an increased risk of premature death. Speech & Language Therapists (SLTs) provide assessment and recommendations to optimise mealtime nutrition and hydration, but little is known about current clinical practice including mealtime recommendation provision and carer adherence support strategies. Before developing any intervention, the context needs to be known.

**Aims:**

This survey aimed to explore the practice of UK SLTs working with school‐aged children with neurodisability and oropharyngeal dysphagia that require mealtime assistance. This included describing workforce and service delivery; assessment practices; mealtime recommendations targeted for example, carer use certain pace, specific utensil; current approaches used to provide mealtime recommendations including the people worked with, delivery modality and adherence support techniques.

**Methods and Procedures:**

An online ethically approved survey was developed using research literature, with SLT stakeholder consultation and piloted prior to dissemination. The survey comprised 36 questions focusing on demographic and caseload information, typical assessment and intervention practice. Questions were multiple choice and free text responses with an upload option for intervention implementation documents. The survey was disseminated using professional networks and social media (summer 2021). Descriptive statistics were used with qualitative analysis for free text and submitted documents.

**Outcomes and Results:**

SLT participants consented and completed demographic and assessment practice questions (*n* = 139) with 102 continuing to the final question. Participants worked across all UK regions, with different employers (NHS, education, independent) and in acute, school and community settings and frequently shared care. Some SLTs had no access to instrumental assessment; videofluoroscopy (*n* = 5, 4%) or FEES (*n* = 88, 63%), and there was limited published assessment use. Participants (*n* = 122) commonly used 17 different mealtime recommendations, most frequently targeting carers to change pace, environment, food consistency (*n* = 120–121, 98%–99%, sometimes‐often). Qualitative analysis identified two practice styles: ‘collaborative creation’ or ‘informative prescription’. 37 SLTs provided 59 intervention documents, (*n* = 39, 66%) were accessible information mealtime mats. There were 28 unique mat templates with 19 different names. Formats and recommendation target frequency differed for example, drink texture (*n* = 27, 96%), pace (*n* = 14, 50%).

**Conclusions and Implications:**

This work provides contextual information on UK SLT practice with school‐aged children with neurodisability and oropharyngeal dysphagia who require mealtime assistance. Further work is required to support research into practice implementation (e.g., assessments), alongside exploration and evaluation of meal mat use and potential SLT consultation styles. These findings contribute to a project creating a resource to improve SLT—family‐carer working.

**WHAT THIS PAPER ADDS:**

*What is already known on this subject*

- SLTs working with children with oropharyngeal dysphagia are typically specialists that work across various settings each week. They, and other health care professionals, report limited use of published assessments but recommend a range of changes which carers need to make within a mealtime (e.g., communicate with their child, make change to food textures, etc.,).

*What this study adds to existing knowledge*

- SLTs have variable time allocated to working with children with oropharyngeal dysphagia while working with diverse children with different diagnoses and needs.
- SLTs frequently share care with other SLTs have limited use of published assessment tools and frequently provide a range of mealtime recommendations requiring carers to change the mealtime.
- SLTs frequently use an accessible ‘mealtime mat’ to provide written recommendations but these are highly variable in name, format and content between services.
- SLTs had two styles when working with family‐carers. They always provide personalised recommendations following assessment, focussing on information provision, but only one style uses fully person‐centred practice.

*What are the potential or actual clinical implication of this work?*

- SLTs would benefit from workforce support to implement evidence‐based practices into their practice when working with children with neurodisability and oropharyngeal dysphagia.
- SLTs could consider using published assessment(s) to enable good shared‐care communication.
- Consideration of which mealtime mats is most effective is needed.
- SLTs may wish to reflect on their personal style—personalised or truly person‐centred.

## 1 Introduction

Children with neurodevelopmental disabilities (neurodisability) have a higher risk of difficulties with eating, drinking and swallowing (Parr et al. 2021) termed ‘oropharyngeal dysphagia’. Oropharyngeal dysphagia may include: ‘*problems with the oral and preparatory phase of swallowing (chewing and preparing the food), oral phase (moving the food or fluid posteriorly through the oral cavity…) and pharyngeal phase (swallowing the food or liquid…)’* (Morgan et al. 2012). Neurodisability includes multiple diagnoses including intellectual disability, Autism and motor disorders such as Cerebral Palsy. All these groups can experience feeding difficulties for example, Cerebral Palsy: 50.4% lifespan prevalence (Speyer et al. 2019). However not all children with neurodisability will have oropharyngeal dysphagia but may fit the wider label of Paediatric Feeding Disorder (PFD) (Goday et al. 2019). PFD is a consensus‐agreed diagnosis that covers four domains: medical, nutritional, feeding skill, and/or psychosocial dysfunction. In the United Kingdom many services are not commissioned to work with children with PFD without oropharyngeal dysphagia (Royal College of Speech & Language Therapists 2023b). This project focuses on children who have oropharyngeal dysphagia due to neurodisability, and who require mealtime assistance (generally with moderate to severe gross motor dysfunction).

Children with neurodisability and oropharyngeal dysphagia who require mealtime assistance for example, need a carer to physically feed or to provide appropriate food texture and so forth., (Sellers et al. 2014), are at greater risk for respiratory infections and hospitalisations (Perez et al. 2017). However, these interventions require parents/carers to be aware of their child's oropharyngeal dysphagia and recognise signs of difficulty including identifying potentially subtle child‐specific signs for example, skin tone change. They also require skills to implement clinicians’ recommendations, typically changing their previous mealtime behaviour. Past research has shown carers may under‐identify oropharyngeal dysphagia (Ball et al. 2012). Callis and colleagues found that parents of children with generalised cerebral palsy and intellectual disability had a low frequency of reported feeding difficulties, suggesting oropharyngeal dysphagia severity tended to be underestimated (Calis et al. 2008). Carer interventions are considered a key priority (Ball et al. 2012) particularly for family‐carers, as even on a school‐day the majority of a child's meals are at home.

Children with neurodisability and oropharyngeal dysphagia need to develop their eating and drinking skills (habilitate) and in the case of acquired conditions, re‐acquire them (rehabilitate). Part of a Speech and Language Therapist's (SLT) role is to assess children's eating, drinking and swallowing function and to provide recommendations to family‐carers to support safe, efficient and enjoyable mealtimes (e.g., carer to change meal by modifying food textures, altering the child's position) (Royal College of Speech & Language Therapists 2023b; National Institute for Health and Care Excellence [NICE] 2017) However, there is little evidence‐based clinical guidance for SLTs on which targets to recommend, how to deliver these recommendations or how to support carers to make these changes.

The Royal College of Speech & Language Therapists (RCSLT) dysphagia clinical guidelines (Royal College of Speech & Language Therapists 2025) specifically considers paediatric dysphagia, which was lacking in the previous guidelines (2015–2025) which covered recommendation targets minimally. The United Kingdom has NICE guidelines regarding the assessment and management of individuals with Cerebral Palsy (NICE 2017). It recommends SLTs should create an individualised plan for managing oropharyngeal dysphagia by assessing and advising on a range of areas including postural management and positioning, modifying fluids and foods, feeding techniques, specialised equipment and more, alongside looking at the knowledge and skills of any person who provides mealtime assistance. This guidance is not specific, for example, modifying food texture could be interpreted as reducing a lumpy texture to a puree or vice versa. Pureeing the texture could be a suitable compensatory strategy to maximise safe eating for a child at risk of choking, however a lumpier texture may be a suitable approach to encourage chewing development (Khamis et al. 2020). There are no other relevant UK NICE guidelines for SLTs to use for other neurodisability groups, for example, Learning Disabilities, Muscular Dystrophy. In this context of limited guidelines, SLT clinical practice for children with oropharyngeal dysphagia may be variable, so an exploration could be beneficial.

### 1.1 Previous Surveys

There is limited evidence related to the clinical practice of SLTs working within PFD including children with oropharyngeal dysphagia. Surveys in the United States have examined the school‐based workforce's confidence and training in managing dysphagia (Wilson et al. 2020; Felicetti et al. 2020). More recent surveys have looked at specific practice aspects for example, telehealth in Australia (Raatz et al. 2020), or practice within low‐income countries (Akhtar et al. 2024).

In the United Kingdom, Pring et al. (2012) explored paediatric SLT clinical working practices including some working in oropharyngeal dysphagia. More recently, a paediatric dysphagia workforce survey (RCSLT 2023b) found differences in service need commissioning and concerns regarding staff recruitment and retention. Finally, the Focus on Early Eating Drinking and Swallowing (FEEDS) Study (Parr et al. 2021) surveyed healthcare and other professionals working with children with eating and drinking difficulties, due to physical or non‐physical causes. They found low published assessment utilisation and identified some common interventions with variable usage reported. However, these surveys did not explore how SLTs work with family‐carers to help them identify or manage their child's eating, drinking and mealtimes.

Chadwick (2017) identified barriers and solutions to dysphagia practice when working with adults with learning disabilities (*n* = 28 SLTs), including education and training of carers and written recommendations. Similarly, Morgan et al. (2018) completed a small survey (*n* = 14 SLTs) investigating the use of accessible documents to provide written mealtime recommendations, sometimes called ‘mealtime advice mats’. Such resources are recommended as a ‘reasonable adjustment’ for people with learning disabilities and dysphagia, with published templates for example, NHS Patient Safety Agency (NPSA) (NHS Patient Safety Agency 2007), although there is limited evaluation of their effectiveness (Royal College of Speech & Language Therapists 2023a).

The limited knowledge of SLT dysphagia practice when working with school‐aged children and their family‐carers alongside the limited and variable guidelines means it is both unclear how SLTs should deliver recommendations to support family‐carers, and how they are managing this complex task presently. Hence an exploration of current practice is required.

### 1.2 Aim and Objectives

The survey aimed to thoroughly explore practice with the following objectives:

1. Describe the UK SLT workforce and service delivery for school‐aged children with neurodisability and oropharyngeal dysphagia who require mealtime assistance, including assessment practices
2. Identify the mealtime recommendations that SLTs target for example, changes carer must make: provide pureed food, position child in particular way
3. Identify the current approaches that SLTs use to provide mealtime interventions including the professionals and carers they work with, delivery modality and adherence support techniques

The survey also explored some additional areas which are reported separately; impact of the COVID‐19 pandemic response (Morgan et al. 2023) and SLTs’ perceived enablers and barriers to their mealtime recommendation practice and family‐carers adherence.

## 2 Materials and Methods

### 2.1 Design and Ethics

This cross‐sectional study delivered an anonymous online national survey of current UK‐based SLT practice. The Checklist for Reporting of Survey Studies (CROSS) (Sharma et al. 2021) guided reporting.

Full ethical approval was granted (ETH2021–2051). Issues considered included: pressure to participate, informed consent, confidentiality/anonymity and data security.

### 2.2 Participants

Participants needed to meet the following inclusion criteria:

‐ Health and Care Professions Council (HCPC) registered SLT
‐ Minimum of 1 year UK‐based clinical experience working with: school‐aged children (Reception to Year 13) with a neurodisability and oropharyngeal dysphagia requiring mealtime assistance

As there are no data available regarding the paediatric dysphagia SLT workforce size, we looked at recent surveys of similar, though broader, workforce groups. These included 103 SLTs working with paediatric dysphagia within a sample of 516 paediatric SLTs (Pring et al. 2012), and 122 working in paediatric dysphagia of 555 SLTs of 17,000 RCSLT members (Royal College of Speech & Language Therapists 2022). We therefore aimed to recruit >100 UK‐based SLTs working with school‐aged children with neurodisability and oropharyngeal dysphagia requiring mealtime assistance.

### 2.3 Survey Development and Content

The survey was drafted by the primary investigator (SM) based on the literature and previous surveys (Chadwick 2017; Raatz et al. 2020; Akhtar et al. 2024) although relevant ones were unavailable during survey development (SPIRIT‐JONES et al. 2018; Parr et al. 2021). Survey questions reflected the UK context for example, RCSLT hubs used for regional reporting, specific stakeholder consultation for questions related to practice enablers, barriers and current Covid‐19 restrictions (Morgan et al. 2023). It was refined through an iterative process in discussion with the research team (KH, KM and KW), stakeholders and further after piloting (see below).

### 2.4 Survey Format

The survey included 36 questions (Supporting Information 1) which included a mixture of multiple choices and Likert scale questions, free text responses, and an option to upload resources. The Participant Information Sheet, eligibility and consent items were displayed within the secure encrypted Qualtrics platform with IP addresses collected to allow participants to return within 2 weeks but not accessed. Specific areas of interest were:

#### 2.4.1 Demographic Information About the SLT Participant and Service

Participant demographic questions aimed to consider the representativeness of the sample for example, region, gender, alongside a range of relevant professional demographic and service provision questions for example, employer, ‘shared care’ with other SLTs. Participants completed multiple option questions indicating aetiologies/conditions associated with PFD and/or oropharyngeal dysphagia representing children in their caseload for example, Cerebral Palsy, genetic conditions including non‐neurodisability causes for example, congenital structural conditions, Avoidant Restrictive Food intake Disorder (ARFID). These questions supported understanding of clinical caseload context, while focussing on children with neurodisability and oropharyngeal dysphagia who need mealtime assistance in subsequent questions.

#### 2.4.2 Assessment Practice

As comprehensive assessment guides individualised intervention and management plans, a range of assessment related questions were included. These explored practice around clinical swallow evaluation and mealtime observation techniques, use of any published clinical observational assessment tools and instrumental assessment accessibility and usage (including the Videofluoroscopic Swallow Study (VFSS)).

#### 2.4.3 Mealtime Recommendations Practice — Engaging With Others

Three questions asked about the people SLTs work with. The Eating and Drinking Assessment Classification Scale (EDACS) (Sellers et al. 2014) was used with permission for participants to outline the level of assistance children on their caseload required, which also enabled a check that participants worked with children needing mealtime assistance. Participants were then asked who they worked with when creating recommendations and who they provided mealtime recommendations to. This final question sent them to two separate branches of questions via forced logic. Participants who stated they worked with parents or family‐carers were routed to questions focusing on working with parents or family‐carers, whether they worked with other professionals or not. Participants who stated they did not work with parents or family‐carers answered the same questions in relation to working with paid‐carers.

##### 2.4.3.1 Mealtime Behaviours Recommended to Family‐Carers

There is no definitive list of the mealtime behaviours that SLTs recommend to family‐carers, therefore a bespoke list was created by SM and KW with reference to different frameworks in the literature, Cochrane review (Morgan et al. 2012), NICE guidelines (2017) and a stakeholder focus group (Morgan and Hilari 2024). This was further reviewed through consultation with the project team and SLT advisors (CH, SJ). The list included behaviours used by carers relevant to different stages of the mealtime. The ‘before mealtime’ question asked participants to rate how often they recommended ten potential mealtime carer behaviour targets; and the ‘during mealtime’ question of 17 different recommendations/targets (See Survey: Supporting Information 1). Examples were given to explain different targets for example, Oro‐motor without swallow—lip and tongue exercises, oro‐sensory with swallow bolus for example, strong tastes—sour, sweet. Additionally, a free text question asked participants to name any specific programmes they might use in practice that is, published approaches or training, including some examples following piloting feedback.

#### 2.4.4 Current Mealtime Recommendations Delivery Modality and Adherence Support Techniques

The final section explored current methods SLTs use to provide mealtime recommendations and support adherence. This included techniques such as giving carers verbal or written information (report and/or mealtime mat) or providing them with training. Participants were provided with a list of recommendations developed from the literature (Charpentier et al. 2020; Chadwick 2017) and asked how frequently (never, rarely, sometimes, often) they used these in their practice. Finally participants were given the opportunity to upload relevant resources they used to deliver mealtime recommendations, for example, mealtime advice mats (Morgan et al. 2018), and alongside potential other written recommendation formats, training manuals or leaflets.

### 2.5 Piloting

The survey was piloted once with *n* = 13 people: eight SLT stakeholders, two SLT Advisors and three members of the research team (KH, KM and KW). There was a purposive attempt to have a diverse stakeholder group with nine (75%) members identifying as female, nine (75%) white, eight (67%) London‐based, seven (58%) in a high NHS band 8a+ role or equivalent, five (42%) clinically active in community and five (42%) in acute settings (some both). The piloting was a final check on question comprehensibility and flow, ensured the survey functioned correctly, for example, accessible via NHS computers, upload function worked, participants could add text for ‘other’ options. Small changes were suggested, outlined above and piloting indicated the survey would take approximately 20 min to complete.

### 2.6 Recruitment

The survey was open from 14th May to 30th July 2021. Participants were recruited via three routes: direct message via specialist professional networks for example, Clinical Excellence Networks (CENs); social media (Twitter (now X), Facebook and LinkedIn); QR code during a short presentation at an online CEN meeting with UK‐wide attendance. All potential participants clicked a link to the Participant Information Sheet and could then progress to consent and the survey.

### 2.7 Analysis

#### 2.7.1 Quantitative Analysis

Relevant questions were downloaded into Microsoft Excel for Office 365 and analysed using suitable descriptive statistics: mean; counts/percentages and endorsement frequencies across categories, for example, never‐rarely versus sometimes‐often responses. Participants could skip questions and so to adapt to missing data statistics were calculated using the participant number for each question.

#### 2.7.2 Qualitative Analysis

The free‐text responses were uploaded into NVivo software to enable qualitative analysis. The answers to the question ‘What do SLTs do to support parents or family‐carers to follow SLT mealtime recommendations?’ were sufficiently rich to be analysed using an inductive qualitative approach, thematic analysis (Braun and Clarke 2006). Free‐text answers expanding on responses to ‘other’ within questions were analysed using a simpler informal content analysis approach, grouping same answers together and synthesising the data when suitable. The uploaded documents were analysed by extracting their data into a spreadsheet using categories for example, title, format, language used, subheadings, mealtime behaviours targeted.

## 3 Results

The main results are presented here and in Supporting Information 2 for a small number of responses, for example, free text answers to ‘other’ option by question.

### 3.1 Survey Response and Progression

Qualtrics recorded 235 arrivals at the landing page, of whom 151 (64%) came to the survey through an anonymous link, 82 were recruited through social media (35%) and 2 (1%) via the QR code. Of the 235, 151 participants (64%) completed the eligibility and consent pages. 1 participant was excluded as they only completed one question. A total of 139 unique IP address survey participants took part beyond the demographic (personal and service) questions, of whom 102 (73%) reached the final question. (Supporting Information 3)

### 3.2 Participant Demographics (Objective 1)

The participants had little diversity (Table 1). They were mostly female, mainly employed by public sector organisations (88%), with the vast majority in the NHS (82%). Most participants were 31–50 years old (69%) and at Band 7 or above (76%). There was a more even spread of some characteristics for example, clinical experience in paediatric dysphagia with the highest percentage of 16+ years (35%), potentially due to being more than a 5‐year span.

**TABLE 1:** Participant demographics.

	Started survey beyond demographics *n *= 139 (%)	Completed final questions *n *= 102 (%)
Gender		
Male	1 (1)	1 (1)
Female	138 (99)	101 (99)
Non‐binary/Prefer not to say	0 (0)	0

Ethnicity		
Ethnic minority or prefer not to say	8 (6)	6 (6)
Prefer not to say	2 (2)	2 (2)
White other	30 (21)	23 (23)
White British	99 (71)	71 (69)

Employer		
Public sector: NHS	115 (83)	84 (82)
Public sector: School/Education	8 (6)	6 (6)
Independent practice	9 (6)	7 (7)
Independent: School/Education	2 (1)	1 (1)
Charity School/Education	3 (2)	2 (2)
Other	2 (1)	2 (2)

Clinical experience in school‐aged children with oropharyngeal dysphagia		
1–2 years	12 (9)	8 (8)
3–5 years	22 (16)	11 (11)
6–10 years	34 (24)	24 (24)
11–15 years	24 (17)	19 (19)
16+ years	47 (34)	36 (35)
**Age bracket**		
21–30 years	17 (12)	10 (10)
31–40 years	52 (37)	38 (37)
41–50 years	40 (29)	32 (31)
51–60 years	28 (20)	20 (20)
60+ years	2 (1)	2 (2)

Banding (or equivalent)		
5	—	1 (1)
6	—	24 (24)
7	—	50 (49)
8a+	—	27 (26)

Disability		
Yes	—	2 (2)
No	—	99 (97)
Prefer not to say	—	1 (1)

UK RCSLT hub region		
East of England	—	7 (7)
East Midlands	—	4 (4)
North East (& Cumbria)	—	9 (9)
North West	—	8 (8)
London	—	24 (24)
South Central	—	2 (2)
South East	—	11 (11)
South West	—	6 (6)
West Midlands	—	5 (5)
Yorkshire & Humber	—	6 (6)
Northern Ireland	—	5 (4)
Scotland	—	7 (7)
Wales	—	8 (8)

*Note*: % Percent rounded to whole number – = Unavailable as questions at the end of the survey (*n* = 38 did not complete).

Abbreviations: NHS = National Health Service, UK = United Kingdom.

### 3.3 Service Provision

Service provision data including work settings, client age ranges and percentage of time providing services to a neurodisability caseload are presented in Table 2. Most participants worked in family homes and schools, with less in acute hospitals. Many SLTs worked across multiple settings (e.g. hospital and community) with the most common ‘other’ (*n* = 21) settings being residential/foster care (*n* = 10, 7%) and respite settings (*n* = 8, 6%). The majority provided services to preschool and school age ranges and provided a variable percentage of time to children with neurodisability and oropharyngeal dysphagia (1‐19%–80‐100%). Most respondents shared care with another SLT (*n* = 97, 69%) both within one service (*n* = 55, 57%) for example, to cover for leave or part‐time work; or across services (*n* = 46, 47%) for example, between independent and public sector or between community and acute teams.

**TABLE 2:** Service provision.

	Number: *n* = 139 (%)
Current work settings^*^	
Acute hospital	36 (26)
Hospital outpatients	33 (24)
Community clinic	47 (34)
School	121 (87)
Home	112 (81)
Other	21 (15)

Age range of clients worked with^*^	
Infants	91 (65)
Pre‐school	125 (90)
School age (Reception‐Year 13)	139 (100)
Adults	33 (24)

Do you share care with another SLT?	
Yes	97 (69)
No	41 (30)
Missing answer	1

Percentage of school‐aged caseload time supporting neurodisability & oropharyngeal dysphagia	
1%–19%	22 (16)
20%–39%	31 (22)
40%–59%	26 (19)
60%–79%	30 (21)
80%–100%	30 (21)

* Items not mutually exclusive. Multiple selections possible.

### 3.4 Clinical Caseload (Objective 1)

Participants worked with a wide variety of children with both neurodisability and non‐neurodisability causes for their PFD (Table 3). Most participants worked with children from varied clinical groups, for example, Cerebral Palsy (*n* = 134, 96%), complex and medically fragile conditions (*n* = 129, 94%), degenerative conditions (*n* = 121, 86%) and all levels of self‐feeding ability (*n* = 123‐137, 88–99%) (Table 3). The most common additionally reported conditions included reflux (*n* = 4) and cancer (*n* = 2) (Supporting Information 2).

**TABLE 3:** Caseload description.

	Number (percentage)
Non‐neurodisability (*n* = 137)*	
Avoidant restrictive food intake disorder	55 (40)
Complex medical and medically fragile conditions	129 (94)
Congenital structural conditions	120 (87)
Metabolic disorders	94 (68)
Unknown aetiology	102 (75)
Other non‐neurodisability conditions	30 (22)

Neurodisability (*n* = 139)*	
Autism spectrum disorder	98 (70)
Cerebral palsy	134 (96)
Neurodevelopmental delay	130 (94)
Developmental conditions (e.g., intellectual disability, down syndrome	137 (97)
Acquired neurological (e.g., childhood stroke, traumatic brain injury)	114 (81)
Degenerative conditions (e.g., Spinal muscular atrophy, muscular dystrophy)	121 (86)
Other neurodisability conditions	14 (10)

EDACS self‐feeding classification (*n* = 138)*	
Independent	123 (88)
Requires assistance	137 (99)
Totally dependent	130 (94)

*: Items not mutually exclusive. Multiple selections possible.

### 3.5 Assessment Practice (Objective 1)

#### 3.5.1 Formal Mealtime or Swallowing Assessment Tools and/or Classification Systems Used (*n* = 101)

Of the 101 respondents who answered this question (Supporting Information 4), many gave non‐answers for example, N/A (*n* = 28, 28%) or reported no formal published tools use (*n* = 9, 9%). Observational frameworks were most commonly reported with locally developed tools (*n* = 15, 15%) or those from textbooks or unpublished courses (*n* = 24, 24%) most frequently used, Multiple other resources for different functions for example, parent‐report, drooling assessment, were reported in small numbers (n ≤ 3). The only other commonly reported tool was a classification system, the EDACS (Sellers et al. 2014) (*n* = 16, 16%).

#### 3.5.2 Instrumental Assessment Access

**FIGURE 1:**
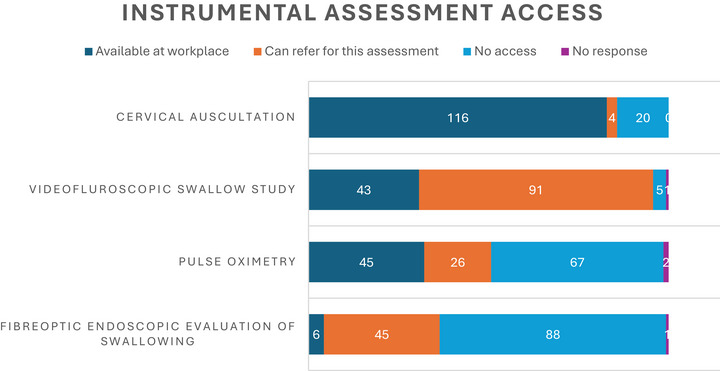
Instrumental assessment access (*n* = 139).

Figure 1 illustrates participants’ access to instrumental assessments of swallowing. The most commonly available on‐site assessment was cervical auscultation (*n* = 116, 83%) however the most accessible (on‐site or via referral) was VFSS (*n* = 134, 96%). The least available was Fibreoptic Endoscopic Evaluation of Swallowing (FEES) (*n* = 51, 36%). A small number of additional instrumental assessments were reported for example, blue dye test for children with tracheostomy tubes (*n* = 2, 1%).

#### 3.5.3 Observational and Instrumental Assessment Usage

Most participants reported regularly completing an observational clinical swallow examination alongside a mealtime observation. When looking at instrumental assessment usage (Table 4) interestingly VFSS was most used (*n* = 107, 81%) despite less on‐site availability than cervical auscultation. Respondents more commonly reported using VFSS ‘sometimes’ (*n* = 83, 63%) rather than ‘often’. FEES usage was very low with only 8% of SLTs who had access to it using it ‘sometimes‐often’ (*n* = 4/51, 8%).

**TABLE 4:** Clinical observational and Instrumental assessment usage (When accessible).

	Never	Rarely	Sometimes	Often
Reported usage of clinical observational assessment (*n* = 139), Number (percentage)				
Clinical swallow examination (oral motor structures, movements and swallow)	3 (2)	3 (2)	22 (16)	111 (80)
Mealtime observation (carer and child mealtime interaction)	1 (1)	1 (1)	5 (4)	132 (95)

Reported usage of instrumental assessments (if have access), Number (percentage)				
Cervical auscultation (*n* = 118)	11 (9)	32 (27)	34 (29)	41(35)
Pulse oximetry (*n* = 70)	7 (10)	31 (44)	23 (33)	9 (13)
VFSS (*n* = 131)	0	25 (19)	82 (63)	24 (18)
FEES (*n* = 51)	24 (47)	23 (45)	2 (4)	2 (4)

### 3.6 Mealtime Recommendations (‘Target Behaviours’) (Objective 2)

Almost all participants provided mealtime recommendations to parent and family‐carers (*n* = 136, 98%) (Supporting Information 5), whilst three branched to answer separate questions focusing on school‐staff only (not reported). Participants (*n* = 53, 38%) suggested a range of other people they provided mealtime recommendations to—most commonly being respite/short break/hospice carers (*n* = 35, 25%) reflecting the level of need of many children with neurodisability and oropharyngeal dysphagia. The following results refer to questions when working with family‐carers.

**FIGURE 2:**
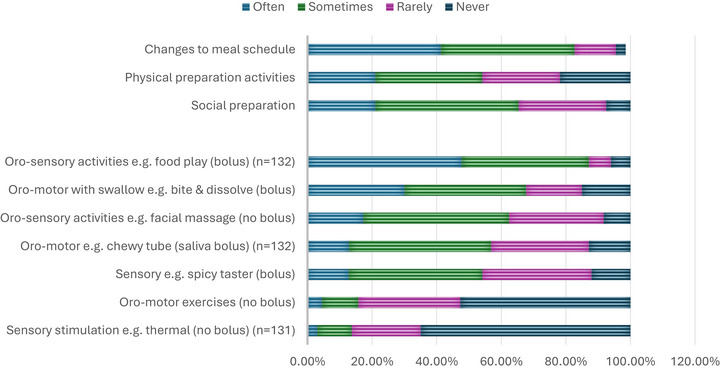
Meal time recommendations usage (before meal) (*n* = 133, unless specified).

In relation to a child's preparation prior to the meal (Figure 2), participants most commonly recommended ‘changes to the meal schedule’ (*n* = 112, 84%, sometimes‐often) with ‘social preparation’ for example, helping the child anticipate the mealtime through a mealtime song and ‘physical preparation’ for example, moving the child into their mealtime chair and space, also recommended at over 50% ‘sometimes‐often’.

Oral‐sensory and motor recommendations generally were more commonly used when a ‘bolus’ was included for example, oro‐sensory activities including a food bolus (*n* = 115, 87%, sometimes‐often), then saliva bolus for example, oro‐motor (*n* = 72, 55%, sometimes‐often) and less frequently ‘no bolus’ recommendations for example, ‘oro‐motor exercises’ (*n* = 21, 16%, sometimes‐often, see Figure 2).

Other suggestions included social mealtime preparation activities for example, washing hands, putting bib on (*n* = 8), specific therapy approaches (*n* = 3), or methods supporting adherence to mealtime recommendations for example, carer training, ‘division of responsibility’ approach (*n* = 10). Further suggestions focused on during the mealtime itself (*n* = 21) for example, environmental changes (*n* = 9), which were also covered within the next survey question ‘during mealtimes’.

**FIGURE 3:**
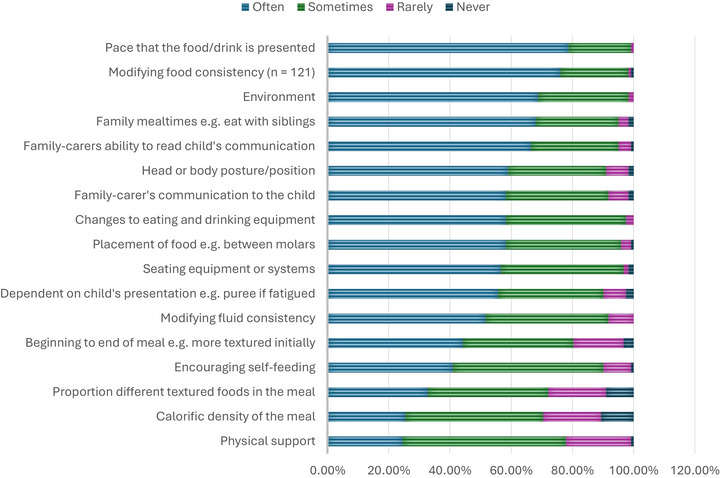
Mealtime recommendation usage (during meal) (*n* = 122, unless specified).

Participants commonly used all recommendations during the meal (Figure 3) with the highest being ‘pace that the food/drink is presented’ (*n* = 121, 99%, sometimes‐often) and the lowest ones were ‘calorific density’ and ‘physical support/assistance’). ‘Other’ recommendations included providing additional explanation of recommendations for example, methods of communication assistance (n = 6, 5%).

#### 3.6.1 Integration of Specific Programmes Into Mealtime Practice or Routines (n = 122)

Most participants stated they did not use any formal or published treatment programmes (*n* = 87, 71%). However, those reporting usage (*n* = 35, 29%) referred to the Sequential Oral Sensory Approach to Feeding (SOS) (*n* = 26, 21%), and less frequently Talk Tools (*n* = 7, 6%) or operant conditioning (*n* = 3, 2%). Some participants suggested they used principles rather than a whole programme (*n* = 8, 7%). There were a range of other programmes reported in use by 1–2 participants with some linked to infant not school‐aged practice (Supporting Information 6).

### 3.7 Mealtime Recommendation Practice (Objective 3)

#### 3.7.1 Delivery Mode of Mealtime Recommendations

**FIGURE 4:**
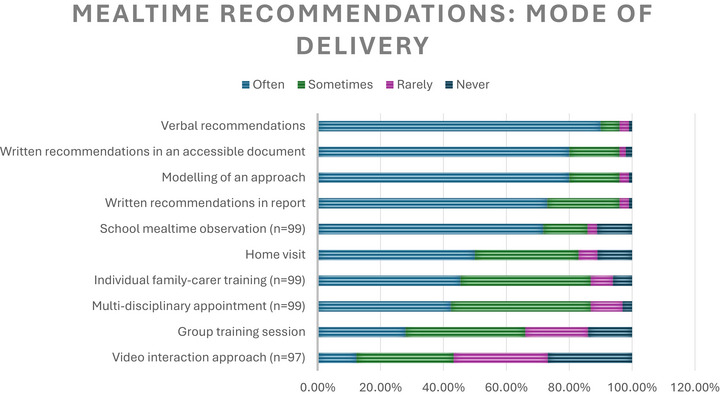
Mealtime recommendations delivery modalities (*n* = 100, unless specified).

A range of delivery modes were in common use (>60% sometimes‐often) to provide and support mealtime recommendation use (Figure 4). The delivery mode choices mainly suggested an ‘informational’ approach (Marques et al. 2020). Both ‘human interactional’ (verbal recommendations) and ‘printed material’ via ‘printed publication’ (written in a report and/or an accessible document) were frequently used. Some indicated the setting for example, home visit, multi‐disciplinary appointment, suggesting ‘face to face’ delivery. Both ‘synchronous’ for example, modelling spoon placement during a meal and ‘asynchronous’ delivery with a ‘printed publication’ and visit/appointment providing information for later implementation were used.

#### 3.7.2 SLTs Description of Adherence Support Techniques

When asked to respond to the free text question ‘*What do you do to support family‐carers to follow SLT recommendations?*’ 118 participants responded with answers varying from a single word to bullet point lists to seven lines of text.

Participants answered this question within one of two consultation styles or typologies that were identified as two themes; ‘*collaborative creation* or ‘*informative prescription’*. Using these styles, they provided support in three sub‐themes linked to the care pathway ‘*recommendation development’*, ‘*recommendation provision’* and ‘*recommendation follow‐up’* with one other sub‐theme *‘school engagement’*. Both styles had some similarities in their approach for example, both discussed the modalities recommendations were provided in. However, the ‘*informative prescribers’* focussed on information provision to support parent understanding and skills whereas the ‘*collaborative creators’* provided information within a broader more holistic approach explicitly starting from parents’ opinions and views (Table 5).

**TABLE 5:** Themes and subthemes with illustrative participant quotes.

	THEMES	
	*Informative prescription*	*Collaborative creation*
*Recommendation development*		AQ.103: Ensure it is a discussion. Find out the parents' concerns…Give parents choices to make a decision.
		AQ.105: Listen to parents concerns discuss the outcome they would like for mealtimes and ways this can be worked towards within the recommendations given. If appropriate gain the views of the child/ young person too.
		AQ.081: I aim to work at ‘where they are’ in terms of their concerns and expectations of SLT support.
*Recommendation provision*	AQ.083: Talk through recs in detail and reasons for same Talk through And make sure they understand the risks of offering food/drinks out with plan.	AQ.093: Problem solve together if not working and go over theory and practical feeding suggestions. Use IDSSI app on iPad to help understand texture levels and use dysphagia app to show swallowing impairment in video firm. Always listen and never judge.
	AQ.066: Explain the importance using simple description or diagrams explain the benefits.	AQ.077: Ensure advice is realistic achievable support locally based SLT to implement recommendations into overall plan ensure the bigger picture is known so that the swallowing/feeding difficulties are considered in context.
	AQ.097: Explain why you are making a change/recommendation Demonstrating/showing/practicing what you want family member to do Provide clear written information alongside verbal instructions so that family can refer back to advice once SLT has left.	AQ.030: .…. Risk assessments/risk feeding protocol as standard so it doesn't feel targeted at particular carers visual guidelines with photos of strategies or important tips such as positioning. Use of you tube videos as reminders for techniques.
*Recommendation follow‐up*	AQ.006: Following this the parents then have my contact details to phone for further support as needed.	AQ.003: Provide multiple sessions to help parents feel confident with recommendations.
	AQ.026: This is challenging due to rates of NRs and these having to be prioritised, therefore reviewing and supporting recommendations takes the hit and we don't always have time to support this.	AQ.130: Involve them throughout and discuss what their concerns are at home child's likes/dislikes at home and how they are managing at home. How best to support them helps them to be part of the process. Videos of their child eating in school and at home so we can all see what is happening. Giving SMART targets to achieve. Follow up with parents regularly to check on the recommendations and any problems they are having.
	AQ.038: A written eating and drinking plan‐ with pictures and different sections. Discuss it with them demonstrate if able or send videos. Sometimes they send me videos to comment on.	AQ.125: Observe. Reflect. Discuss. Explain. Model. Support and encourage. Problem solve. Review. Reflect on progress.
	AQ.073 Model strategies provide written recommendations regular reviews to model new strategies. Offer training/workshops.	AQ.069: I am able to offer frequent follow up/review appointments—ongoing long term support and encouragement seems vital‐ managing expectations.
*School engagement*	AQ.011: Train them to follow the plan‐ session with school staff to talk through the plan at a mealtime. Offer the same with parents or discuss over the phone.	AQ.060: Support families with providing training/ awareness to schools—particularly to mainstream schools—often families feel they are being difficult and need professional support to get their voice heard in schools.
	AQ.094: Copy school based plans home when updated.	AQ.092: Open up honest discussion about preferences routine challenges at home/school and safety.

##### 3.7.2.1 Recommendation Development

Participants taking a ‘*collaborative creation’* approach frequently discussed ‘*recommendation development’*, with co‐creation of recommendations based on families’ concerns and priorities as part of assessment, ‘See parents as experts’ (AQ.076). However, this sub‐theme was not evident in respondents taking an ‘*informative prescription*’ approach, who described assessment within ‘*recommendation provision’*.

##### 3.7.2.2 Recommendation Provision

Various aspects were described as part of ‘*recommendation provision’* with much similarity between both participant theme approaches. There was a focus on providing both verbal and written recommendations, often including a mealtime advice mat format, under both themes. Participants outlined a focus on parents understanding of the SLT's rationale (swallowing process, risk, consequences) and on skill development (modelling, training). Many participants also described the importance of accessible information. However, those following ‘*collaborative creation’* had a more discursive, negotiated or gradual recommendation provision approach. The word ‘discuss(ion)’ was used by participants in both styles but within ‘*informative prescription’* the sense was of information provision and persuasion to follow the SLT or Multidisciplinary Team (MDT) expert recommendations, whereas in ‘*collaborative creation’* parents’ views could clearly be the key deciding factor.

##### 3.7.2.3 Recommendation Follow‐Up

Respondents then discussed ‘*recommendation follow‐up*’. This was often during a description of providing a ‘review’. This could be either face‐to‐face at home or school or remote follow‐up via phone or video call. Those in the ‘*informative prescription’* style discussed reviews in terms of monitoring adherence, providing feedback or further information and were more likely to leave the follow‐up to parental discretion. Those in the ‘*collaborative creation’* approach anticipated that family‐carers may not have followed the recommendations for multiple reasons and expected an ongoing dialogue about them.

##### 3.7.2.4 School Engagement

Interestingly despite the question focusing on parents and family‐carers, some participants focussed on school staff. Sometimes this was working with both together or how a child differed between settings. Other times, participants (all being informative prescribers), focused on school‐staff alone.

No distinguishing demographic factor (e.g., employer, setting, work experience length) was evident to explain which collaboration style the SLT utilised.

#### 3.7.3 Resource Documents Used

Participants (*n* = 37/102, 36%) provided *n* = 59 written resources used when supporting family‐carers to carry out mealtime recommendations. Some provided multiple documents (*n* = 22, 37% from 7 participants). A range of documents were uploaded, with the majority, *n* = 39 (66%) being mealtime mats, alongside report templates (*n* = 4, 7%), care plan templates (*n* = 3, 5%), topic advice sheets (*n* = 8, 14%) for example, thickeners, IT guidance about sharing videos via a portal, a school staff feeding competencies record (*n* = 1, 1%) and recommendations lists (*n* = 2, 3%).

There were 28 unique mealtime mat templates with the others being different examples by need for example, child with oral intake versus nil‐by‐mouth, or mealtime assistance/supervision level required. There were 19 different names between the 28 templates for example, Eating & Drinking /Feeding/ Mealtime Advice/Guidelines/Information/Plan. Four mats (14%) clearly used an adapted version of the NPSA (2007) template, and four (14%) others potentially did. Formats differed (*n* = 18, 64% landscape, *n* = 26, 93% colour, *n* = 28, 100% in sections) as did language (*n* = 23 (82%) third person language, *n* = 18 (64%) used colour‐coded risk level and content (*n* = 20 (71%) provided SLT contact details). The most common recommendation targets related to ‘Food texture’ (*n* = 26, 93%) and ‘Drink texture’ (*n* = 27, 96%) with *n* = 27 (96%) using IDDSI descriptors (Cichero et al. 2017), ‘Position’ (*n* = 25, 89%), Carer's ability to read child (*n* = 22, 79%) with some less evident for example, ‘Pace’ (*n* = 14, 50%), or unable to be determined.

#### 3.7.4 Professional and Carer Engagement

**FIGURE 5:**
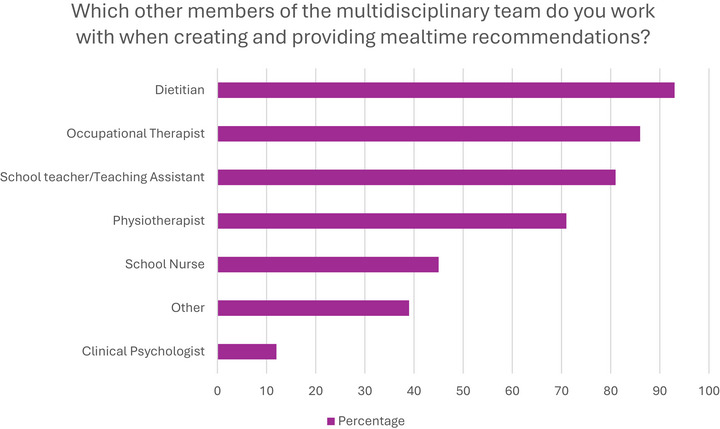
Multidisciplinary team members (*N* = 132).

Participants reported frequently working with several Multidisciplinary Team (MDT) members (Figure 5, Supporting Information 2) when creating and providing mealtime recommendations, most commonly dietitians (*n* = 123, 93%), closely followed by occupational therapists (*n* = 114, 86%) and with psychology team members rare (*n* = 16, 12%).

Participants frequently worked with school education staff (*n* = 107, 81%), but less often with school nurses *n* = 59, 45%). A large number (*n* = 52, 39%) of respondents listed other professionals. They most commonly highlighted the role of parent/carers within the MDT (*n* = 24, 18%), alternate nursing roles for example, ward (*n* = 7, 5%) and a wide range of medical specialities (*n* =  29, 5%).

## 4 Discussion

This is the first survey to have thoroughly examined the practices of UK SLTs working with school‐aged children with neurodisability and oropharyngeal dysphagia. SLTs reported limited use of formal published assessments or interventions. However, they regularly provided multiple mealtime recommendation targets advising on activities both prior to and during the meal. Providing recommendations via an accessible mealtime mat was common. Participants seemed to utilise two main styles when supporting family‐carer adherence to recommendations: ‘*collaborative creation’* or ‘*informative prescription’*. Mealtime mats were frequently used to communicate recommendations but were variable in nature including their name and only a small number used a previously published template (NHS Patient Safety Agency 2007).

### 4.1 SLT Workforce

Our survey of over 100 SLTs working with school‐aged children with neurodisability and oropharyngeal dysphagia has a comparable response rate to previous surveys including UK SLTs working in paediatric dysphagia, *n* = 102 (Pring et al. 2012), *n* = 131, (Parr et al. 2021), *n* = 183 (RCSLT 2023b). Responses were received from all UK regions and reflect the geographical spread for example, more in London and South East, of other surveys, (*n* = 3755) (Health & Care Professions Council 2021) including the paediatric dysphagia workforce (RCSLT 2023b). Overall, this indicates this survey is representative of the UK paediatric dysphagia SLT population. Demographic diversity comparison with other previous high‐income country SLT surveys is limited due to non‐collection/reporting of these demographics (Raatz et al. 2020; Spirit‐Jones et al. 2018; Felicetti et al. 2020). However, in comparison to the Health and Care Professions Council's (HCPC) survey data (HCPC 2021) our study participants are slightly less diverse than the profession generally for example, gender (99% female, HCPC: 96%), ethnicity (92% white, HCPC: 89%) and disability (99% no disability, HCPC: 92%). It will be important to ensure increased SLT student workforce diversity becomes evident in this clinical setting over time.

The high number of participants at specialist Band 7 or above reflects the workforce, where SLTs working in paediatric dysphagia are more likely to be specialists than in other paediatric clinical areas (Pring et al. 2012; RCSLT 2023b). Both studies also, like ours, found SLTs were regularly working across multiple settings.

### 4.2 Service Delivery

Shared care was commonly evident including shared cases between SLTs unlikely to have access to shared notes, as working in different teams/services. This could indicate that a shared framework/language when supporting families with mealtime recommendations may be beneficial. Within dysphagia services and research, shared terminology approaches have been targeted and widely adopted recently, with aims to improve communication and consistent care, for example, IDDSI framework (Cichero et al. 2017). This survey's results' regarding assessment and recommendation practice, discussed below, indicates a shared language between SLTs may be lacking and needs to be considered.

Many SLTs are working with children across multiple settings, with children at different ages and with a wide range of reasons for oropharyngeal dysphagia, not solely neurodisability. This aspect of their role was sometimes for a small caseload or amount of time/week as also found within other countries (Raatz et al. 2020; Wilson et al. 2020; Akhtar et al. 2024). As reported recently (Royal College of Speech & Language Therapists 2023b) some participants stated they were not commissioned to work with children with feeding difficulties that are not attributed to oropharyngeal dysphagia for example, Autism. However, others were clearly working with children who meet the wider criteria of PFD (Goday et al. 2019) alongside oropharyngeal dysphagia. This provides a challenge for SLTs to keep up to date in all paediatric dysphagia management areas when compared with those working with adults, who may be more likely to specialise in a client, group/condition for example, head and neck cancer, stroke. For example, different types of neurodisability present with different clinical patterns of dysphagia and will require different mealtime recommendations for example, children with neuromuscular disease have more difficulties with solids than liquids whereas this is typically reversed in children with Cerebral Palsy (Van Den Engel‐Hoek et al. 2011; Arvedson 2013). Clinical guidelines, resources and training for SLTs need to consider these differences, alongside the need to encourage thorough assessment prior to identifying mealtime recommendation targets.

### 4.3 Assessment Practice

The participants reported limited standardised assessment usage, which was consistent with practice in Australia and New Zealand, when compared with US practice (Spirit‐Jones et al. 2018). The FEEDS survey (Parr et al. 2021) found small numbers reporting published assessment and outcome measure usage despite their questions supporting recall by specifically naming assessments. They found a higher participant number suggesting they used the Therapy Outcome Measures (TOMS) (Enderby and John 2019) (13%) compared to only one in this study.

Recent reviews of the psychometric characteristics of paediatric dysphagia non‐instrumental assessments have found many to be lacking (Speyer et al. 2018). Interestingly the one assessment recommended by Speyer et al. (2018), the Dysphagia Disorders Survey (Sheppard et al. 2014), was not listed by participants or reported in 2018 (Parr et al. 2021) despite being a highly relevant tool, focussing on people with intellectual disabilities from two years to adults. This highlights the difficulty of getting research evidence into clinical practice, with structural barriers a strong likely contributing factor as the assessment previously required on‐site training not available in the United Kingdom, although now available online. One tool reported in more common use was the EDACS (Sellers et al. 2014). Usage was also reported by Parr et al. (2021) (5% usage of EDACS and another measure). Our findings might suggest increased usage over time with 12% usage (16 of 136 participants working with children with Cerebral Palsy). These findings indicate the importance of considering implementation of research findings thoroughly as this participant group has not adopted published tools widely. It is concerning considering the frequency of shared‐care evident in this survey, that SLTs will not necessarily have shared language and measures to communicate about a child's needs.

Encouragingly, the majority of SLTs reported having access to instrumental assessment options. Many suggest instrumental methods are necessary for a complete assessment considering the need to observe aspects of the swallow not visible during clinical observations, particularly the pharyngeal phase and visual confirmation of (silent) oropharyngeal aspiration (Garand et al. 2020). Children with neurodisability and oropharyngeal dysphagia are likely to meet the criteria for a VFSS (Royal College of Speech & Language Therapists 2025). NICE (2017) also recommend that VFSS assessment may be necessary for example, to determine recommendation effectiveness, review anatomical changes during puberty. Therefore, the most common ‘sometimes’ use of VFSS may warrant further investigation and interestingly SLTs have reported issues with consistent instrumental assessment access (RCSLT 2023b). In relation to the low use of FEES, Spirit‐Jones et al. (2018) also found this within Australian practitioners compared with US counterparts. The RCSLT (2025) suggest this method is particularly suitable for infants, and so the school‐aged focus of this survey may explain why use was lower despite availability. As discussed above, the need for thorough assessment, including instrumental tools, to help direct recommendation target selection will need to be considered.

The most accessible on‐site instrumental assessment was cervical auscultation but was less commonly used. Recent research has found cervical auscultation improves detection of features of aspirating and non‐aspirating swallows (Frakking et al. 2016), while barriers to uptake may include access to suitable training.

### 4.4 Mealtime Intervention Practice — Recommendation Targets

Our study found that changes to the meal schedule and oro‐sensory food play were the most common targets recommended prior to the meal. There was a low usage of oro‐motor exercises (no bolus) for example, ‘never’ used by 53% of participants (*n* = 71), alongside a greater focus on motor learning feeding interventions, with oro‐motor with swallow (bolus) usage by 86% (*n* = 114). This is encouraging as it matches the research evidence base suggesting motor learning feeding interventions, with functional activities matching the task, that is, having a food bolus, are the best option, with limited research evidence for oro‐motor exercises that have less specificity to the task (Khamis et al. 2020; Novak et al. 2020). Felicetti et al. (2020) found overall US oro‐motor exercise practice (*n* = 44 (55%) of *n* = 77 for example. targeting range of motion, coordination), although whether these were delivered using a motor learning approach or without a bolus was not specified.

For mealtime recommendations during the meal, SLTs suggested use of all 19 items. There is currently limited research‐evidence base for many of these (Morgan et al. 2026) but they reflect those reported by others (Parr et al. 2021). Parr's team (2021) however found lower use of recommendations than this study, for example 43% (*n* = 179) of Health Care Professionals reported pacing intervention use, compared to 99% (*n* = 121) of our participants. By focussing solely on SLTs this survey identified that SLTs create and provide the verbal and written recommendations, and recommend multiple mealtime behaviours more frequently than reported by the whole MDT (Parr et al. 2021). This difference was evident for other targets; only 56% (*n* = 237) targeting food or drink modification, whereas high use for food (*n* = 120, 99%) and fluids (*n* = 122, 100%) in our study. This may be when not within a professional group's expertise for example, a physiotherapist, deferring to the SLT. We also found the recommendations least used by SLTs linked to other professions’ remit for example, calorific density, physical support.

This survey's focus on SLT mealtime recommendations meant that predominately feeding skill and somewhat nutrition and psychosocial needs were addressed but the PFD domain of medical dysfunction (Goday et al. 2019) was not explored. However, this survey considered a greater number of mealtime recommendations in‐depth alongside frequency. For example, food modification was often used by 76% (*n* = 92) versus fluid modification often used by 52% (*n* = 63) of SLTs. This separation of texture modification into food and drink is beneficial considering the current debate regarding fluid thickening as an appropriate intervention (Stewart and Burr 2021).

Our finding that multiple recommendations are being targeted is reflective of the complexity of these children and raises the question of how SLTs prioritise what and how many aspects to change. The free text responses suggested two SLT practice styles. The ‘*collaborative creation’* style whereby SLTs actively engage with the family‐carers to prioritise what to change and gradually add other targets, whereas SLTs using the ‘*informative prescription’* style self‐directed priority setting with multiple targets. Both approaches were personalised to the child for example, assessment‐driven person‐specific advice, but only the ‘*collaborative creation’* approach could be described as person‐centred care.

### 4.5 Mealtime Intervention Practice — Approaches in Use

SLTs worked with a wide range of professionals when creating and providing mealtime recommendations. Interdisciplinary working is recommended as good holistic practice; however parents can have mixed thoughts on their experiences of such teams (Cowpe et al. 2014). Parr's team (2021) surveyed a wider range of professionals and family‐carers to come to a final set of suggested interventions. Any future developments to support mealtime recommendation practice will need to consider this wider professional team but also the specific differences between each professions practice. The survey also found that SLTs provide recommendations to multiple mealtime assistant roles that may have differing knowledge, skills and values, such as paid respite‐carers and school staff. However, in some free text comments there was a suggestion that there is not much differentiation between home and school recommendations. This will need further exploration as to whether approaches developed for SLTs and family‐carers could be used with others that provide mealtime assistance or vice versa.

The approaches that SLTs used to support mealtime recommendation adherence in the multi‐choice question commonly reflected those within their free text answers: verbal and written instruction, classified as an ‘informational’ delivery mode (Marques et al. 2020). Written recommendation practice has been reported in other countries including the US, with seven of 133 participants recommending them for a hypothetical case (Felicetti et al. 2020), and in New Zealand, with an exploration of inconsistent Eating Drinking & Swallowing Plans use within one school (Miles et al. 2021). Informational modalities focus on knowledge and skill development alongside accessible information specifically, and reflect previous findings and recommendations within adult learning disability research (Chadwick 2017). As SLTs have specialist skills in working with people with communication needs, this may be a professional bias influence. In adult dysphagia an explanatory model of adherence has been suggested (Krekeler et al. 2020; Krekeler et al. 2018) with some work within the area of head and neck cancer (Govender et al. 2017) using implementation science frameworks for example, Behaviour Change Techniques Taxonomy (Michie et al. 2013) to consider a wider range of barriers to adherence and strategies to support. Exploration using such theoretical models within paediatric dysphagia is lacking though some is emerging (Morgan et al. 2026). ‘Face to face’ delivery was described (home visit, multidisciplinary appointment) alongside ‘distance’ delivery for example, telehealth (Morgan et al. 2023; Raatz et al. 2020), whether this has continued in the UK post‐pandemic restrictions would be beneficial to explore. The most commonly provided written resource used by SLTs was mealtime mats. However, there was considerable variety in format, language and content with the recommendation areas not fully matching the SLT reported recommendation usage in other questions. This is the first known study to examine these physical resources. It is unknown which mat may be most effective in what situations and there is a need for effectiveness evaluations (RCSLT 2023a).

A smaller number of participants, those following a ‘c*ollaborative creation’* approach, suggested additional approaches beyond ‘informational’ provision for example, co‐creation and discussion rather than direct explanation and ensuring understanding. The lowest reported approach on the Likert scale was ‘video interaction approach’, potentially the most collaborative intervention, where the carer is videoed providing a meal and then reviews it with the SLT to consider strengths and potential areas to change, for example Parent Child Interaction Therapy (McNeil and Hembree‐Kigin 2011). Resources to support a fully collaborative discussion with family‐carers may be warranted. However, as a change in practice for some SLTs careful consideration is needed on how this can be successfully implemented. Use of implementation science approaches within this field to both improving carers adherence to recommendations and how to change SLT practice would be beneficial.

### 4.6 Strengths and Limitations

The survey was carefully considered using the evidence base and stakeholder engagement and used a mixed methods approach. It provides a wide‐ranging description of current UK SLT practice when working with children with neurodisability and oropharyngeal dysphagia who require mealtime assistance, including assessment, mealtime recommendation targets and approaches to support family‐carers adherence. The participant numbers and regional spread suggest it is representative of the current UK SLT population and context. It adds to a limited knowledge of SLT practice in paediatric dysphagia in general and could support future exploration of potential global similarities and differences (Spirit‐Jones et al. 2018; Akhtar et al. 2022). It also demonstrates that practice differs between SLTs and the wider professional groups (Parr et al. 2021).

There are some limitations to consider. The participants worked across a variety of age ranges, client groups and settings and some did not respond solely regarding school‐aged children with neurodisability and oropharyngeal dysphagia practices who need mealtime assistance for example, suggested pre‐school or tracheostomy assessments, neonatal interventions. Although the participants appear representative of previous UK surveys (e.g., regional and gender diversity), limited workforce data prevents determining some representativeness aspects for example, acute versus community workforce, and whether participation bias was present.

In response to SLT stakeholder feedback, the two open text questions where participants listed published assessment tools and intervention programmes, some examples were included. The given examples were most frequently listed, with limited novel responses. This may be due to participants aiming to please and so reporting usage of tools they do not use. Interestingly, despite providing names of multiple assessments Parr et al. (2021) also found low published tool usage in line with our findings.

To reduce choices and maintain survey progression, some options were broad with examples given but no detailed definition. For example, ‘modifying food consistency for example, puree meals’ could mean reducing (compensatory) or increasing (skill development) food textures. In addition, one question item in our study had a misleading example. This may explain its exception to the trend of more frequent use of recommendations that included bolus use than interventions without a bolus. The item ‘Oral sensory without swallow’ had ‘teething toys’ as an example. This could be interpreted as stimulation to the lips and mouth that is, ‘with swallow of a saliva bolus' with the results then matching the trend.

Finally, the qualitative analysis is reliant on solely one question. An interview approach could have yielded a more in‐depth response. However, the analysis did identify two distinctive themes of clinical approach which strongly resonated during SLT and family‐carer engagement and involvement. In addition, these qualitative findings reflect the quantitative results, providing support for data trustworthiness and this method captured the views of a larger number of participants when compared with an interview methodology.

## 5 Conclusion

This survey has outlined the practice of UK SLTs working with school‐aged children with neurodisability and oropharyngeal dysphagia. These SLTs are typically specialists who work across multiple settings and with children with a wide range of diagnoses, some for a limited time, and who frequently share care. This creates a challenge for SLTs to keep up to date with the research evidence base. It is evident that the transfer of published assessments and interventions into practice is low. Most SLTs have access to one ‘gold standard’ instrumental assessment, within a hospital, with use common, but more usually used ‘sometimes’. Cervical auscultation is under‐used considering its accessibility and an exploration of its benefit in paediatric community settings could be warranted.

Despite limited clinical guidelines there was often agreement regarding the usage of a wide range of mealtime recommendations and the modality that they, and any support strategies for adherence, are delivered. These reports indicate SLTs are the key professionals creating and discussing mealtime recommendations with family‐carers, despite this being in a context of multidisciplinary work. There is a focus on knowledge and skill development of family‐carers with information accessibility seen as key. Considering the limited report of research‐practice implementation in this group, regarding formal assessment tool usage, and the differing approaches taken to support mealtime recommendation adherence, further exploration of current practice using an implementation science approach would be beneficial.

## Funding

The primary investigator was funded through a Barts Charity Nursing/Allied Health Professionals Clinical Doctoral Fellowship (G‐001829). Barts Charity did not have input into the survey's design, implementation or analysis.

## Ethics Statement

This survey received formal ethical approval by City St George's, University of London Language and Communication Sciences Proportionate Review Ethics Committee (ETH2122‐2120).

## Consent

This study did not recruit patients. The SLT participants provided consent both to take part in this anonymous survey and provided optional consent for free text comments usage in publications. One participant declined consent for free text comments usage with their data analysed but not quoted.

## Conflicts of Interest

The authors declare no conflicts of interest.

## Supporting information




**Supporting Information**: jlcd70254‐supp‐0001‐SuppMat.pdf

**Supporting Information**: jlcd70254‐supp‐0002‐SuppMat.pdf

**Supporting Information**: jlcd70254‐supp‐0003‐SuppMat.pdf

**Supporting Information**: jlcd70254‐supp‐0004‐SuppMat.pdf

**Supporting Information**: jlcd70254‐supp‐0005‐SuppMat.pdf

**Supporting Information**: jlcd70254‐supp‐0006‐SuppMat.pdf

## Data Availability

Supplementary information, including data analysis is provided. The anonymous survey data will be made available through the primary author's City St George's, University of London open data repository space via FigShare, following thesis submission.
