# Supplementary material for: Working With School‐Aged Children With Neurodisability and Oropharyngeal Dysphagia Who Require Mealtime Assistance: A Survey of Speech and Language Therapists’ Clinical Practice
Source: Int J Lang Commun Disord. 2026 Apr 29;61:e70254. doi: 10.1111/1460-6984.70254 (PMC13129504; doi:10.1111/1460-6984.70254)
Supplement: Supplementary file 1 — Supporting Information: jlcd70254‐supp‐0001‐SuppMat.pdf [file JLCD-61-0-s005.pdf]

Participant Info Sheet and consent

Participant Information Sheet ETH2021-0996 20.1.2021 V.2

Speech & Language Therapists clinical practice for school-aged children with neurodisability and oropharyngeal dysphagia who need carer mealtime assistance - a survey.
(Part of the SEEM study*)

Principal researcher Sally Morgan PhD student
Supervised by: Professor Katerina Hilari, Dr Kathleen Mulligan & Dr Kelly Weir

We invite you to take part in a research study. Before you decide whether you would like to take part, it is important that you understand why the research is being done and what it would involve for you. Please take time to read the following information carefully and discuss it with others if you wish.
If you have any questions, or would like more information before making your decision you can contact Sally Morgan on sally.morgan.2@city.ac.uk. You can download a copy of this information to keep here

What is the purpose of the study?
This survey seeks to gather the views of speech and language therapists about usual clinical practice when providing dysphagia intervention and mealtime recommendations for school-aged children with neurodisability and oropharyngeal dysphagia who need carer mealtime assistance. There are questions about the dysphagia and mealtime recommendations SLTs make, the barriers that family carers experience in following these recommendations and what helps to overcome these barriers.

This survey is being conducted as part of Sally Morgan's doctoral studies. The results will be used to create a 'best-practice toolkit' for SLTs to support children and their families to improve children's mealtime safety, efficiency and enjoyment.

Why have I been invited to take part?
We are looking for HCPC registered practicing speech and language therapists who work in the clinical area of school-aged children with neurodisability and oropharyngeal dysphagia. They need to have worked in the UK and working with children with neurodisability and oropharyngeal dysphagia who need carer mealtime assistance, for a minimum for 12 months.

Do I have to take part?
No. Participation in the project is voluntary, and you can choose not to participate in part or all of the project. Once you have completed the survey, you will not be able to withdraw your answers. However, you can choose to leave the survey when you wish. Any answers you have provided to that point will be retained and analysed.

What will happen if I take part?
You can read this Participant Information and then decide to take part or not.
You are invited to complete an online survey lasting about **20-30 minutes**. There will be a mixture of questions. There is an **opportunity** to **upload an example document** used to support mealtime recommendations e.g., information sheets, training resources.

If you wish to, **prepare** a **resource before you start** the survey. You will be able to part-complete the survey and return within 2 weeks to finish it if you have cookies enabled, use the same device and the same web browser when you return using the anonymous link. You will be able to return up to 2 weeks from starting the survey. Ideally **plan a convenient time** to complete the survey.

What are the possible disadvantages and risks of taking part?
We do not foresee any risks in taking part in an online survey. You are accessing an anonymous

link and there are no personally identifiable questions. Qualtrics collects IP addresses to allow you to partially complete and return a survey for up to 2 weeks, but these details are not provided to the researchers in the standard results and will not be accessed by the researchers.

If you choose to upload an example document that is not anonymised the primary researcher will ensure the document is de-identified before being used for analysis. If you provide a free text quote that could identify you then it will be anonymised before being analysed.

What are the possible benefits of taking part?
There are no direct benefits of taking part, except for a **chance to reflect on your clinical practice**. This project is part of a larger research project which aims to create a ‘best-practice toolkit’ for SLTs to support children and families improve children’s mealtime safety, efficiency and enjoyment.

How is the project being funded?
This project is funded by Barts Charity through an Allied Health Professionals Clinical Doctoral Fellowship awarded to Sally Morgan (the principal researcher).

Data privacy statement
City, University of London is the sponsor and the data controller of this study based in the United Kingdom. This means that we are responsible for looking after your information and using it properly. The legal basis under which your data will be processed is City’s public task.

Your right to access, change or move your information are limited, as we need to manage your information in a specific way in order for the research to be reliable and accurate. To safeguard your rights, we will use the minimum personal-identifiable information possible (for further information please see https://ico.org.uk/for-organisations/guide-to-data-protection/guide-to-the-general-data-protection-regulation-gdpr/lawful-basis-for-processing/public-task/).

Survey respondents may opt to obtain survey results by entering their email addresses at the end of the survey via a separate link. These emails will not be connected with survey responses. The only people at City who may have access to this identifiable information will be Sally Morgan & Professor Katerina Hilari. City will keep identifiable information about you from this study for up to 1 year after the survey has closed to send a summary copy of the project’s results.

You can find out more about how City handles data by visiting https://www.city.ac.uk/about/governance/legal. If you are concerned about how we have processed your personal data, you can contact the Information Commissioner’s Office (IOC) https://ico.org.uk/.

What will happen to the results?
The survey responses and documents uploaded will be analysed. Results of the survey will contribute to the principal researcher’s doctoral thesis and may be shared via conference presentations, publication in a peer-reviewed journal, dissemination at events for people with neurodisabilities, their families and speech & language therapists. If you add free text responses, we may use quotations when reporting the results. These will not be identifiable to you or your service.

If you would like to receive a summary of the project’s results then you can provide your email address via the separate link at the end of the survey.

After completion of the doctoral thesis and/or publication in a peer-reviewed journal the anonymous data will be uploaded to the University’s open data repository following good data management principles. The anonymous survey responses and de-identified uploaded documents may be used for secondary data analysis projects in the future. This will not occur without additional ethical approval.

Who has reviewed the study?
This study has been approved by City, University of London Language & Communication Science Proportionate Review Research Ethics Committee.

What if there is a problem?
If you have any problems, concerns or questions about this study, you should ask to speak to a member of the research team. If you remain unhappy and wish to complain formally, you can do this through City’s complaints procedure. To complain about the study, you need to phone 020 7040 3040. You can then ask to speak to the Secretary to Senate Research Ethics Committee and inform them that the name of the project is ‘Speech & Language Therapists clinical practice for school-aged children with neurodisability and oropharyngeal dysphagia who need carer mealtime assistance - a survey’.

You can also write to the Secretary at:
Anna Ramberg
Research Integrity Manager
City, University of London, Northampton Square
London, EC1V 0HB
Email: Anna.Ramberg.1@city.ac.uk

Further information and contact details
You can contact Sally Morgan sally.morgan.2@city.ac.uk in the first instance or Professor Katerina Hilari K.Hilari@city.ac.uk with any enquiries about the research.

Thank you for taking the time to read this information sheet.

*The SEEM study: Safe, Efficient and Enjoyable Mealtimes: Creating a toolkit for families of children who need assistance with eating and drinking - a multi-method investigation.

SLT clinical practice for school-aged children with neurodisability and oropharyngeal dysphagia who need carer mealtime assistance - a survey. (Part of the SEEM study*)

Remember
The survey has **28** questions, with 8 additional questions related to Covid19 and to explore participant diversity (**36**).

You may wish to ensure you have **20-30 minutes** available to take part in this survey, or you can leave and return using the same device, browser and with cookies enabled for up to 2 weeks from starting the survey.

You may wish to **prepare any anonymised resources/documents** you are willing to share e.g., mealtime guidelines, dysphagia training manual, before you start the survey and **complete** this survey using the device they are stored on e.g., **PC/laptop.**

*The SEEM study: Safe, Efficient and Enjoyable Mealtimes: Creating a toolkit for families of children who need assistance with eating and drinking - a multi-method investigation.

Eligibility to take part

Thank you for agreeing to take part in the survey.

To be eligible you must be able to select all of the options below.

I confirm that:

- [ ] 1. I am a HCPC registered Speech & Language Therapist
- [ ] 2. I have a MINIMUM of 1 year UK based clinical experience working with:
- [ ] SCHOOL-AGED children (Reception to Year 13) with NEURODISABILITY
- [ ] AND OROPHARYNGEAL DYSPHAGIA
- [ ] who also need CARER MEALTIME ASSISTANCE

Consent

○ I give consent to take part in the survey

○ I do not give consent to take part in the survey

Consent to use your free text answers when sharing the results

○ I give consent for anonymised quotes to be used in any sharing of survey results

○ I do not give consent for anonymised quotes to be used in any sharing of survey results (you can still complete the survey)

You, service and caseload

This survey is exploring the typical practice and management recommendations of SLTs when supporting children with neurodisability and oropharyngeal dysphagia around mealtimes.

Although COVID-19 may have changed elements of your current practice, we ask you to think holistically about how you would approach this typically i.e., NOT during Covid-19. There are a few questions at the end about potential changes in practice due to the Covid-19 pandemic.

This first set of questions ask about you, your service and your caseload

Q.1 What is your gender?

- ◯ Male
- ◯ Female
- ◯ Non-binary
- ◯ Prefer not to say

Q.2 What is your ethnicity?

- ◯ Asian
- ◯ Asian British
- ◯ Black
- ◯ Black British
- ◯ Mixed or multi-ethnic
- ◯ White
- ◯ White British
- ◯ Other ethnic background
- ◯ Prefer not to say

Q.3 What age bracket do you fall within?

- ◯ 21- 30
- ◯ 31-40
- ◯ 41-50
- ◯ 51-60
- ◯ 60+

Q4. What age range of clients do you work with?
Select all that apply

- ☐ Infants (0 - 1;0 years)

- ☐ Pre-school (>1;0 - 4;11 years, including UK Nursery)
- ☐ School age (Reception-Year 13)
- ☐ Adults (18 years plus)

Q5. How many years of experience do you have working with school-aged children with neurodisability and oropharyngeal dysphagia?

- ○ 1-2 years
- ○ 3-5 years
- ○ 6-10 years
- ○ 11-15 years
- ○ 16 years +

Q6. Who is your main employer?

- ○ Public sector – NHS
- ○ Public sector – School/Education
- ○ Independent – School/Education
- ○ Charity – School/Education
- ○ Independent practice
- ○ University
- ○ Other (please specify)

Pediatric feeding disorder info

Goday et al. (2019) define a new umbrella term 'Pediatric Feeding Disorder' to be "Impaired oral intake that is not age-appropriate", and is associated with:

***Medical* (aspiration) AND/OR**

***Nutritional* (malnutrition or nutritional deficiency) AND/OR**

***Feeding skill* (need for texture modification, modified feeding strategies) AND/OR**

***Psychosocial* (avoidance of when feeding or being fed, disruption of caregiver-child relationship associated with feeding) dysfunction.**

Q.7a Which client groups do you see of children with 'pediatric feeding disorder'?
Select all that apply
NON-NEURODISABILITY

- [ ] Avoidant Restrictive Food Intake Disorder (ARFID)
- [ ] Complex medical and medically fragile conditions (e.g., cardiac conditions, chronic lung disease, including tracheostomy in situ)
- [ ] Congenital structural conditions (e.g., cleft lip/palate, craniofacial conditions, other syndromes and sequences that affect structures)
- [ ] Metabolic Disorders
- [ ] Unknown aetiology
- [ ] Other non-neurodisability conditions (please list all)

Q.7b Which client groups do you see of children with 'pediatric feeding disorder'?
Select all that apply
NEURODISABILITY

- [ ] Autism Spectrum Disorder
- [ ] Cerebral Palsy
- [ ] Neurodevelopmental delay
- [ ] Developmental conditions (e.g., intellectual disability, down syndrome, other syndromes or sequences that affect development)
- [ ] Acquired neurological (e.g., childhood stroke, traumatic brain injury)
- [ ] Degenerative conditions (e.g., Spinal muscular atrophy, Muscular Dystrophy)

☐ Other neurodisability conditions (please list all)

Children with 'pediatric feeding disorder' may not always have oropharyngeal dysphagia.

Q.8 In which settings do you manage school-aged children with oropharyngeal dysphagia and neurodisability?

Select all that apply

☐ School
☐ Home
☐ Community clinic
☐ Hospital outpatients
☐ Acute Hospital
☐ Other settings (please describe)

Q.9 Do you ever share a school-aged child's dysphagia care with another SLT?

○ No
○ Yes (If so, please describe)

Q. 10 What percentage of your school-aged caseload involves supporting those with neurodisability and oropharyngeal dysphagia needs?

- 1-19%
- 20-39%
- 40-59%
- 60-79%
- 80-100%

Assessment practice

We would like to know a little about your assessment practice

Q. 11 How frequently do you use these assessment approaches?

	Never	Rarely	Sometimes	Often
Clinical Swallow Examination (assessment of oral motor structures, movements and swallow etc.)	○	○	○	○
Mealtime observation (assessment of carer-child mealtime interaction)	○	○	○	○

Q. 12 Which of these instrumental assessments are available to you?

	Available at workplace	Not available at workplace but can refer for this assessment	No access to this instrumental assessment
Cervical auscultation	○	○	○
Pulse oximetry	○	○	○
Videofluroscopic swallow study (VFSS)	○	○	○
Fibreoptic Endoscopic Evaluation of Swallowing (FEES)	○	○	○

	Available at workplace	Not available at workplace but can refer for this assessment	No access to this instrumental assessment
Other (please name)	○	○	○
Additional (please name)	○	○	○

How frequently do you use/refer for cervical auscultation assessment?

- Never
- Rarely
- Sometimes
- Often

How frequently do you use/refer for pulse oximetry assessment?

- Never
- Rarely
- Sometimes
- Often

How frequently do you use/refer for VFSS assessment?

- Never
- Rarely
- Sometimes
- Often

How frequently do you use/refer for FEES assessment?

- Never

- ◯ Rarely
- ◯ Sometimes
- ◯ Often

Q. 13 Please list any formal mealtime or swallowing assessment tools and/or classification systems that you use with school-aged children e.g. Eating & Drinking Ability Classification System (EDACS), Jay's observational assessment of dysphagia?

[ ]

Children with neurodisability and oropharyngeal dysphagia may need different levels of physical assistance at mealtimes.

Q. 14 What level of feeding support do the children with neurodisability and oropharyngeal dysphagia on your caseload require?

Question based on Eating and Drinking Ability Classification Scale (Sellers at al., 2013) with permission

Select all that apply

- ☐ Independent – able to bring food & drink to their mouth without any assistance (may need another to modify food texture, may be unable to sit independently)
- ☐ Requires assistance – need help to bring food & drink to their mouth, either from another person or through the use of adapted equipment
- ☐ Totally dependent – totally dependent upon another person to bring food or drink to the mouth

Q. 15 Which other members of the multidisciplinary team do you work with when creating and providing mealtime recommendations?
Select all that apply

- ☐ Physiotherapist
- ☐ Occupational Therapist
- ☐ Dietitian
- ☐ School Nurse
- ☐ Clinical Psychologist
- ☐ School Teacher/Teaching Assistant

☐ Other (please specify)

Q. 16 Which carers do you provide mealtime recommendations to?
Select all that apply

☐ Parents/family-carers

☐ School staff/carers

☐ Hospital staff/carers

☐ Other (please describe)

Mealtime support level & family carer interventions

The remaining questions focus on children with neurodisability and oropharyngeal dysphagia who require mealtime assistance and the work you do to support **Parents or FAMILY-Carers.**

Q. 17 When providing recommendations to parents or family-carers to improve the safety, efficiency and enjoyment of mealtimes, how frequently do you target the following?

Recommendations **just before the meal**:

	Never	Rarely	Sometimes	Often
Oro-motor with swallow bolus practice e.g., Mendelsohn manoeuvre, biting practice (bite & dissolve transitional foods),	○	○	○	○
Sensory with swallow bolus practice e.g., strong tastes – sour, sweet, cold	○	○	○	○

	Never	Rarely	Sometimes	Often
Oro-motor with saliva bolus practice e.g., chewing pouch, chewy tube	O	O	O	O
Oro-motor without swallow e.g., lip and tongue exercises	O	O	O	O
Sensory without swallow e.g., thermal tactile stimulation	O	O	O	O
Oro–sensory activities with swallow bolus e.g., desensitisation to reduce aversion to food (food play and may bring to lips)	O	O	O	O
Oro-sensory activities without swallow e.g., facial massage, teething toys	O	O	O	O
Physical preparation activities e.g., placing hands on table/tray, encouraging to stamp feet	O	O	O	O
Social preparation e.g. social story about the meal, visual timetable that meal is soon	O	O	O	O
Changes to meal schedule e.g. ensure oral meal is an hour after a tube feed	O	O	O	O
Other (1) (please specify) [ ]	O	O	O	O
Other (2) (please specify) [ ]	O	O	O	O
Other (3) (please specify) [ ]	O	O	O	O

Recommendations **during the meal**:

	Never	Rarely	Sometimes	Often
Changes to head or body posture/position e.g., chin tuck	○	○	○	○
Changes to seating equipment or systems e.g., tilt chair	○	○	○	○
Physical support e.g., place finger on chin to encourage lip closure	○	○	○	○
Modifying fluid consistency e.g., thicken drinks	○	○	○	○
Modifying food consistency e.g., puree meals	○	○	○	○
Changes to calorific density of the meal e.g., add butter to mashed vegetables	○	○	○	○
Changes to eating and drinking equipment e.g., different spoon, cup	○	○	○	○
Changes to placement of food e.g., to side of mouth to encourage chewing	○	○	○	○
Encouraging self-feeding e.g., hand over hand support	○	○	○	○
Changes to the environment e.g., noise level, turning off tv	○	○	○	○
Family mealtimes e.g., eating with siblings/parents	○	○	○	○
Changes to the family-carer's communication to the child e.g., describing the food, offering choice of what food next	○	○	○	○
Changes to the family-carers ability to read communication from the child e.g., cue based feeding	○	○	○	○
Changes to pace that the food/drink is presented e.g., give them a break after 3 spoonfuls	○	○	○	○
Changes from beginning to end of meal e.g., more difficult textures initially and then a puree for pudding/Timing of different foods presented (relative to fatigue levels)	○	○	○	○
Proportion of foods which are of different textures in the meal e.g., easy to swallow foods vs. chewable foods	○	○	○	○
Changes dependent on child's presentation e.g., if recently had a seizure then lower level of texture	○	○	○	○

	Never	Rarely	Sometimes	Often
Other (1) (please specify)	○	○	○	○
Other (2) (please specify)	○	○	○	○
Other (3) (please specify)	○	○	○	○

Q 18 Do you have specific programmes that you integrate into mealtime practice or routines e.g., Talk tools, SOS, operant conditioning?

- ○ No
- ○ Yes (If so, please list)

We would like to know what helps/hinders when providing mealtime recommendations.

Q. 19 What do you do to support parents or family-carers to follow SLT mealtime recommendations?
Please describe:

Q. 20 What barriers or difficulties do you think parents or family-carers face when carrying out SLT mealtime recommendations?
Please describe:

Q. 21 From a parent or family-carers perspective, what other factors do you think enable them to carry out SLT mealtime recommendations?
Please describe:

Q 22 When providing recommendations to improve the safety, efficiency and enjoyment of mealtimes to parents or family-carers which of the following methods do you use

	Never	Rarely	Sometimes	Often
Verbal recommendations	○	○	○	○
Written recommendations in report	○	○	○	○
Written recommendations in an accessible document e.g., mealtime guidelines/mealtime assistance mat	○	○	○	○
Verbal explanation of why the recommendations are required e.g., risk of aspiration, impact on nutrition	○	○	○	○
Home visit	○	○	○	○
School mealtime observation	○	○	○	○
Multi-disciplinary appointment e.g., Dietitian and SLT together	○	○	○	○
Group training session e.g., general information on dysphagia	○	○	○	○

	Never	Rarely	Sometimes	Often
Individual family-carer training e.g., bespoke 1-1 session	○	○	○	○
Modelling of an approach e.g., how to thicken a drink, pacing should use	○	○	○	○
Video interaction approach	○	○	○	○
Other (1) (please describe)	○	○	○	○
Other (2) (please describe)	○	○	○	○
Other (3) please describe)	○	○	○	○

Q.23 Thinking about your own <u>knowledge</u>, <u>training</u>, <u>skills</u>, and <u>experience</u> please tell us about <u>factors</u> that <u>help you</u> to provide effective SLT mealtime recommendations for <u>parents</u> or <u>family</u>-carers.

Q.23a Thinking about your own <u>knowledge</u>, <u>training</u>, <u>skills</u>, and <u>experience</u> please tell us about <u>factors</u> that <u>make it difficult</u> to provide effective SLT mealtime recommendations for <u>parents</u> or <u>family</u>-carers.

Q.24 Thinking about <u>peer</u>, <u>service</u> or <u>organisational</u> <u>factors</u> please tell us what <u>helps you</u> to provide effective SLT mealtime recommendations for <u>parents</u> or <u>family</u>-carers.

Q.24a Thinking about <u>peer</u>, <u>service</u> or <u>organisational</u> <u>factors</u>, please tell us about anything that <u>makes it difficult for you</u> to provide effective SLT mealtime recommendations for <u>parents</u> or <u>family</u>-carers.

Q.25. Please describe your <u>thoughts, feelings and opinions</u> about providing SLT mealtime recommendations for <u>parents</u> or <u>family</u>-carers.

Q. 26 Please describe <u>anything you</u> would <u>like to change</u> about your SLT mealtime recommendation practice.

Q.27 Are there <u>any other aspects</u> that <u>support or hinder</u> you when providing SLT mealtime recommendations for <u>family</u>-carers?

Q. 28 We would like to analyse the content of resources you use to support family-carers to carry out SLT mealtime recommendations. Please upload here a copy of any such documents e.g., report templates, mealtime guidelines, training manuals.

Please anonymise (no client case information, remove organisational logos). All documents will be checked and de-identified before being analysed.

You can leave the survey now and return for up to 2 weeks to upload the documents. However in order to return you will need to:
- Use the same device
- Use the same browser
- Have cookies enabled

Upload Resource 1:

Upload Resource 2:

Upload Resource 3:

Covid-19 practice

This survey asked about your typical practice. We are also interested in the **impact Covid-19** has had **on school-aged children's dysphagia management.**

Q. 29 Covid-19 has impacted my school-aged children's dysphagia management clinical practice

Strongly disagree ○ | Somewhat disagree ○ | Neither agree nor disagree ○ | Somewhat agree ○ | Strongly agree ○

Q. 30 What Covid-19 factors negatively impacted your delivery of school-aged children's dysphagia management?
Select all that apply

- ☐ School absences due to closures, reduced offer of places
- ☐ Personal re-deployment so not managing this caseload
- ☐ Redeployment of other colleagues so larger caseload
- ☐ Redeployment of other members of the multidisciplinary team
- ☐ Unable to visit child at home e.g., due to family shielding, social isolation of a family member etc.
- ☐ Impact of face coverings/masks on assessment
- ☐ Personal barriers in using telehealth e.g., skills in IT and new systems, lack of resources (correct software, access to wifi etc.)
- ☐ Family barriers in using telehealth e.g., lack of resources (IT equipment/smart phone, wifi and data), skills in IT
- ☐ Family members contracted Covid19, unwell and unable to attend appointments
- ☐ Family declined input as reduced capacity while managing other priorities due to Covid19 e.g., all children home from school
- ☐ Some children on caseload contracted Covid19 and affected their eating, drinking and swallowing skills
- ☐ Other (please specify)

Q. 31 What Covid-19 factors positively impacted your delivery of school-aged children's dysphagia management?
Select all that apply

- ☐ Increased personal access to telehealth resources as part of role to allow virtual home visits
- ☐ Increased access of families to telehealth resources
- ☐ Increased personal skills in telehealth

- ☐ Increased family-carer skills in telehealth
- ☐ Increased focus on home intervention as children at home, rather than school
- ☐ Other (please specify)

Q.32 What learning or changes in clinical practice initiated by the response to Covid-19 are you likely to maintain?
Please give details:

Additional demographics

Four final additional questions about yourself, to check that survey participants represent the diversity of your profession

Q 33 Do you describe yourself as having a disability?

- ○ Yes
- ○ No
- ○ Prefer not to say

Q. 34 What Agenda for Change band is your role (or equivalent in on a different contract)

- ○ Band 5
- ○ Band 6
- ○ Band 7
- ○ Band 8a +

Q. 35 What is your job title?

Q36. What is the MAIN part of the UK/RCSLT hub you work in?

- Northern Ireland
- Scotland
- Wales
- England – North East & Cumbria
- England – North West
- England – East of England
- England – East Midlands
- England – West Midlands
- England – London
- England – South East
- England – South Central
- England – South West
- England – Yorkshire & Humber

Carer branch barriers and enablers

The remaining questions focus on children with neurodisability and oropharyngeal dysphagia who require mealtime assistance and the work you do to support **CARERS (School, hospital or other).**

Q. 17 When providing recommendations to improve the safety, efficiency and enjoyment of mealtimes, how frequently do you target the following?

Recommendations **just before the meal**:

	Never	Rarely	Sometimes	Often

	Never	Rarely	Sometimes	Often
Oro-motor with swallow bolus practice e.g., Mendelsohn manoeuvre, biting practice (bite & dissolve transitional foods),	☐	☐	☐	☐
Sensory with swallow bolus practice e.g., strong tastes – sour, sweet, cold	☐	☐	☐	☐
Oro-motor with saliva bolus practice e.g., chewing pouch, chewy tube	☐	☐	☐	☐
Oro-motor without swallow e.g., lip and tongue exercises	☐	☐	☐	☐
Sensory without swallow e.g., thermal tactile stimulation	☐	☐	☐	☐
Oro–sensory activities with swallow bolus e.g., desensitisation to reduce aversion to food (food play and may bring to lips)	☐	☐	☐	☐
Oro-sensory activities without swallow e.g., facial massage, teething toys	☐	☐	☐	☐
Physical preparation activities e.g., placing hands on table/tray, encouraging to stamp feet	☐	☐	☐	☐
Social preparation e.g. social story about the meal, visual timetable that meal soon	☐	☐	☐	☐
Changes to meal schedule e.g. ensure oral meal is an hour after a tube feed	☐	☐	☐	☐
Other (please specify) [text box]	☐	☐	☐	☐
Other (2) (please specify) [text box]	☐	☐	☐	☐

	Never	Rarely	Sometimes	Often
Other (2) (please specify)	☐	☐	☐	☐

Recommendations **during the meal**:

	Never	Rarely	Sometimes	Often
Changes to head or body posture/position e.g., chin tuck	☐	☐	☐	☐
Changes to seating equipment or systems e.g., tilt chair	☐	☐	☐	☐
Physical support e.g., place finger on chin to encourage lip closure	☐	☐	☐	☐
Modifying fluid consistency e.g., thicken drinks	☐	☐	☐	☐
Modifying food consistency e.g., puree meals	☐	☐	☐	☐
Changes to calorific density of the meal e.g., add butter to mashed vegetables	☐	☐	☐	☐
Changes to eating and drinking equipment e.g., different spoon, cup	☐	☐	☐	☐
Changes to placement of food e.g., to side of mouth to encourage chewing	☐	☐	☐	☐
Encouraging self-feeding e.g., hand over hand support	☐	☐	☐	☐
Changes to the environment e.g., noise level, turning off tv	☐	☐	☐	☐
Social mealtimes e.g., eating with classmates/parents	☐	☐	☐	☐
Changes to the carer’s communication to the child e.g., describing the food, offering choice of what food next	☐	☐	☐	☐
Changes to the carers ability to read communication from the child e.g., cue based feeding	☐	☐	☐	☐

	Never	Rarely	Sometimes	Often
Changes to pace that the food/drink is presented e.g., give them a break after 3 spoonfuls	☐	☐	☐	☐
Changes from beginning to end of meal e.g., more difficult textures initially and then a puree for pudding/Timing of different foods presented (relative to fatigue levels)	☐	☐	☐	☐
Proportion of foods which are of different textures in the meal e.g., easy to swallow foods vs. chewable foods	☐	☐	☐	☐
Changes dependent on child's presentation e.g., if recently had a seizure then lower level of texture	☐	☐	☐	☐
Other (1) (please specify) ______	☐	☐	☐	☐
Other (2) (please specify) ______	☐	☐	☐	☐
Other (3) (please specify) ______	☐	☐	☐	☐

Q 18 Do you have specific programmes that you integrate into mealtime practice or routines e.g., Talk tools, SOS, operant conditioning?

○ No

○ Yes (please list)

We would like to know what helps/hinders when providing mealtime recommendations.

Q. 19 What do you do to support carers to follow SLT mealtime recommendations?
Please describe:

Q. 20 What barriers or difficulties do you think carers face when carrying out SLT mealtime recommendations?
Please describe:

Q. 21 From a carer's perspective, what other factors do you think enable them to carry out SLT mealtime recommendations?
Please describe:

Q 22 When providing recommendations to improve the safety, efficiency and enjoyment of mealtimes to carers which of the following methods do you use

	Never	Rarely	Sometimes	Often

	Never	Rarely	Sometimes	Often
Verbal recommendations				
Written recommendations in report				
Written recommendations in an accessible document e.g., mealtime guidelines/mealtime assistance mat				
Verbal explanation of why the recommendations are required e.g., risk of aspiration, impact on nutrition				
Home visit				
School mealtime observation				
Multi-disciplinary appointment e.g., Dietitian and SLT together				
Group training session e.g., general information on dysphagia				
Individual Carer training e.g., bespoke 1-1 session				
Modelling of an approach e.g., how to thicken a drink, pacing should use				
Video interaction approach				
Other (1) (please describe)				
Other (2) (please describe)				
Other (3) (please describe)				

Q.23 Thinking about your own <u>knowledge</u>, <u>training, skills</u>, and <u>experience</u> please tell us about <u>factors</u> that <u>help you</u> to provide effective SLT mealtime recommendations for <u>carers.</u>

Q.23a. Thinking about your own <u>knowledge</u>, <u>training</u>, <u>skills</u>, and <u>experience</u>, please tell us about <u>factors</u> that <u>make it difficult</u> for you to provide effective SLT mealtime recommendations for <u>carers.</u>

Q.24 Thinking about <u>peer</u>, <u>service</u> or <u>organisational</u> <u>factors</u> please tell us what <u>helps you</u> to provide effective SLT mealtime recommendations for <u>carers</u>.

Q.24a Thinking about <u>peer</u>, <u>service</u> or <u>organisational</u> factors, please tell us about anything that <u>makes it difficult</u> for you to provide effective SLT mealtime recommendations for <u>carers</u>.

Q.25. Please describe your <u>thoughts, feelings and opinions</u> about providing SLT mealtime recommendations for <u>carers.</u>

Q. 26 Please describe anything you would like to change about your SLT mealtime recommendation practice.

Q.27 Are there any other aspects that support or hinder you when providing SLT mealtime recommendations for carers?

Q. 28 We would like to analyse the content of resources you use to support carers to carry out SLT mealtime recommendations. Please upload here a copy of any such documents e.g., report templates, mealtime guidelines, training manuals.

Please anonymise (no client case information, remove organisational logos). All documents will be checked and de-identified before being analysed.

You can leave the survey now and return for up to 2 weeks to upload the documents. However in order to return you will need to:

- Use the same device
- Use the same browser
- Have cookies enabled

Upload Resource 1:

Upload Resource 2:

Upload Resource 3:

Powered by Qualtrics
