# Supplementary material for: Working With School‐Aged Children With Neurodisability and Oropharyngeal Dysphagia Who Require Mealtime Assistance: A Survey of Speech and Language Therapists’ Clinical Practice
Source: Int J Lang Commun Disord. 2026 Apr 29;61:e70254. doi: 10.1111/1460-6984.70254 (PMC13129504; doi:10.1111/1460-6984.70254)
Supplement: Supplementary file 2 — Supporting Information: jlcd70254‐supp‐0002‐SuppMat.pdf [file JLCD-61-0-s002.pdf]

Supporting Information 2: ‘Other’ responses within the survey

Table 1 Participant Demographics Responses for ‘other’

Employer:

Local Authority: n = 1

Private employer: n = 1

Table 2 Service Provision Responses for ‘other’

Other work settings (n=21) (sometimes multiple):

Respite/short breaks: n = 10

Residential care/Foster home care: n = 5

Hospice: n = 3

Nursery/Preschool: n = 3

Charity commissioned services: n = 2

Videofluroscopy: n = 1

Inpatient (medium secure & forensic): n = 1

Shared care examples:

More than 2 SLTs in a service share care e.g., for specialist opinion, for full cover due to leave etc., to mentor: n = 55

Between acute/tertiary & community SLT: n = 33

Between independent & public sector SLTs: n = 9

Between community services e.g., child lives in different borough to school: n = 4

No shared care but have supervision: n = 4

Did not answer: n = 3

Table 3 Caseload Description Responses for ‘other’

Other Non-neurodisability conditions (n = 22):

Gastroenterology: n = 2 (with isolated feeding issue/to exclude dysphagia)

Reflux: n = 4

Surgical: n = 1

Oncology: n = 2

Restricted eating (may not have formal ARFID diagnosis: n) = 1

Selected 'unknown' and gave an example: Idiopathic: n = 3

Selected 'structural' but gave examples Tracheoesophageal fistula (TOF): n = 3, Upper airway anomalies: n = 1, Laryngomalacia: n = 1

Sensory impairments: n = 1

Feeding difficulties 'no matter what diagnosis': n = 1

Gave examples of neurodisability conditions:

Cerebral Palsy: n = 1

Developmental: n = 3

Acquired Brain Injury: n = 1

Peripheral neuromuscular conditions/ Spinal Muscular Atrophy: n = 2

Prematurity/Neonates: n = 5

Lissencephaly: n = 1

Dystonia: n = 1

Other neurodisability conditions (n = 11):

ASD only if have not yet developed safe oropharyngeal skills (no commissioning for ARFID or sensory feeding difficulties): n = 2

Oncology: n = 1

Mitochondrial disorder: n = 1

No response: n = 3

Selected 'developmental conditions' but gave examples of rare/genetic conditions e.g. MPSIII, Pitts-Hopkins, KMT2B mutation, Rett: n = 5

Selected 'acquired neurological' but gave examples: brain tumour, encephalitis: n = 2

The following additional examples were given by participants when providing 'other' responses to non-neurodisability conditions (previous question) but did not select the corresponding relevant choice in the neurodisability question:

Peripheral neuromuscular conditions/ Spinal Muscular Atrophy: n = 2

Prematurity/Neonates: n = 5

Lissencephaly: n = 1

Dystonia: n = 1

Figure 1: Instrumental Assessment Access Other responses

Incentive spirometer: n = 1
Barium swallow: n = 1
Respiratory exam: n = 1
Baseline assessment for Expiratory Muscle Strength Training: n = 1

Figure 2: Recommendations given before the meal

Other recommendations also given:
Other (1) (n = 62) Gave a suggestion: n = 33
Other (1) (n = 57) Gave a suggestion: n = 18
Other (1) (n = 51) Gave a suggestion: n = 12

Sensory warm up/preparation (similar to physical preparation): n = 4
Oral hygiene: n = 1
Parent feeder's positioning: n = 2
More examples of 'social preparation' e.g., object of reference, mealtime song, inclusion in meal preparation (setting table, smelling food), washing hands, neckerchief/bib on: n = 8
Deeper explanation e.g., would not use facial massage but would recommend teething toys, would recommend some strategies separate from mealtime: n = 2

Suggestions that were offered in next 'during meal' recommendation question within survey:
Changes to environment: n = 9
Changes to positioning of child: n = 7
Equipment: n = 3
Family meal: n = 1
Different presentations e.g., seizure: n = 1

Suggestions that are related to assessment and recommendation adherence:
Parent-carer training, Easy read document, a Talking Mat (Murphy, 1998) to discuss likes-dislikes, a culture of 'division of responsibility' (Satter, 2022): n = 10
Specific therapy approaches e.g. SOS (Toomey and Ross, 2011), Facial-Oral Tract therapy (FOTT) (Coombes, 2008): n = 3

Figure 3: Recommendations given during the meal

Other recommendations also given:
Other (1) (n = 54) Gave a suggestion: n = 19
Other (1) (n = 50) Gave a suggestion: n = 15
Other (1) (n = 49) Gave a suggestion: n = 8

Parent feeder's positioning = 2
Offering new foods with a preferred food = 3
How to wipe/clean face = 1
Sensory aspects of food & drink e.g., carbonation = 1
Dependent on setting e.g., different positioning at school & home (different equipment) = 2
Division of responsibility (Satter, 2022) = 1

Further explanation of selected targets e.g.
Placement of food (centrally) = 2

Family mealtimes (family style serving) = 1
Pacing (drink at beginning or end or after X spoonfuls) = 1
Communication (objects of reference, symbols, offering choices, meal mat) = 6
Child’s position (using tray) = 1
Self-feeding (backward chaining, active participation e.g., waiting for lip closure) = 1

Figure 5 Multidisciplinary team members ‘other’ responses:

Child: n = 1
Parent/Carers: n = 24
Consultant/Community Paediatrician: n = 19
Community/Paediatric Learning Disability/Specialist school nurse: n = 4
Ward nurses/HCAs: n = 3
Acute medical team: n = 5
Medical specialities: Neurology: n =2/Gastroenterology: n = 1/Respiratory: n =1/Ear Nose & Throat (ENT): n = 1
Surgeon: n = 1
Respite carers: n = 3
Specialist teacher e.g., Visual Impairment: n= 1
SENDCo: n = 1
Educational Psychologist: n = 1
Oral health worker: n = 1
Play workers: n = 1
Residential carers: n = 1
No response: n = 84

References:

COOMBES, K. 2008. FOTT Facial-Oral Tract therapy. *IADH Magazine. International Association for Disability and Oral Health*.
MURPHY, J. 1998. Talking Mats: speech and language research in practice. *Speech and Language Therapy in Practice*, 11-14.
SATTER, E. 2022. *Division of Responsibility in Feeding* [Online]. Available: https://www.ellynsatterinstitute.org/how-to-feed/the-division-of-responsibility-in-feeding/ [Accessed].
TOOMEY, K. A. & ROSS, E. S. 2011. SOS approach to feeding. *Perspectives on Swallowing and Swallowing Disorders (Dysphagia),* 20, 82-87.
