# Supplementary material for: Working With School‐Aged Children With Neurodisability and Oropharyngeal Dysphagia Who Require Mealtime Assistance: A Survey of Speech and Language Therapists’ Clinical Practice
Source: Int J Lang Commun Disord. 2026 Apr 29;61:e70254. doi: 10.1111/1460-6984.70254 (PMC13129504; doi:10.1111/1460-6984.70254)
Supplement: Supplementary file 3 — Supporting Information: jlcd70254‐supp‐0003‐SuppMat.pdf [file JLCD-61-0-s001.pdf]

Supporting Information 3: Participant response numbers and progression through the survey flow diagram

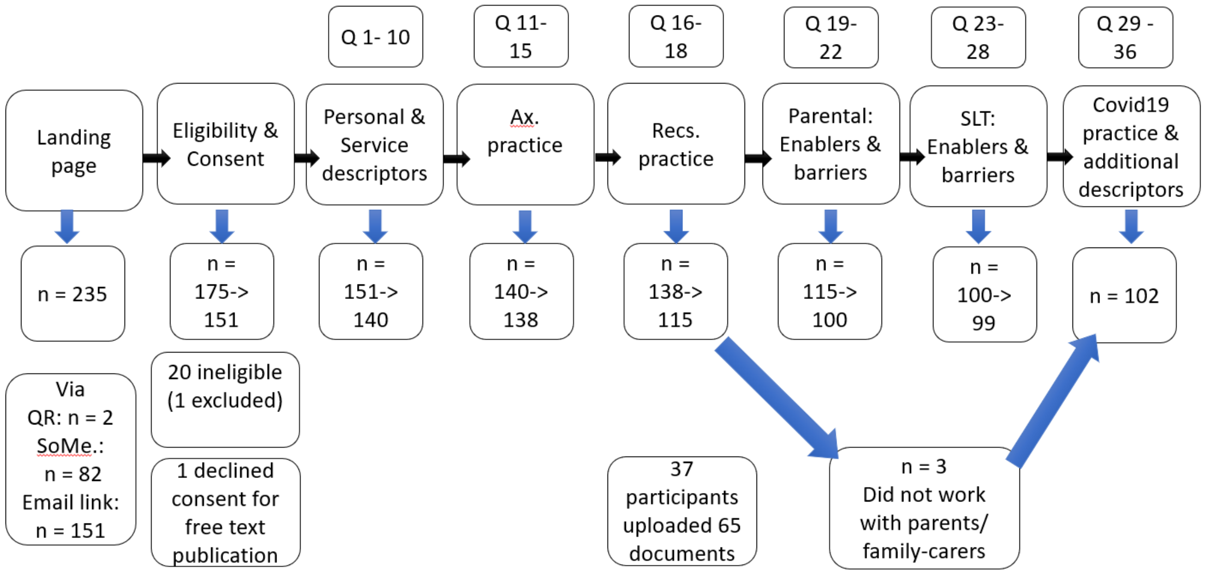

Figure: Participant response numbers and progression through the survey

Key: Ax.= Assessment, Recs. = Recommendations, SLT = Speech & Language Therapist, SoMe = Social Media
