# Supplementary material for: Working With School‐Aged Children With Neurodisability and Oropharyngeal Dysphagia Who Require Mealtime Assistance: A Survey of Speech and Language Therapists’ Clinical Practice
Source: Int J Lang Commun Disord. 2026 Apr 29;61:e70254. doi: 10.1111/1460-6984.70254 (PMC13129504; doi:10.1111/1460-6984.70254)
Supplement: Supplementary file 4 — Supporting Information: jlcd70254‐supp‐0004‐SuppMat.pdf [file JLCD-61-0-s004.pdf]

Supporting Information 4: Formal mealtime or swallowing assessment tools &/or classification systems (n=101)

	Number (Percentage)
No response given	
Not answered	28 (28)
None or N/A	9 (9)
Observational assessment frameworks	
Jay's observational assessment (Hibberd, 2001)	24 (24)
Locally/personally created tool	15 (15)
Template from Alex Kelly's book (Kelly, 2001)	1 (1)
Template for course attended led by Sue Strudwick	1 (1)
Schedule of Oral Motor Assessment (SOMA) (Skuse et al., 1995)	2 (1)
Parent-report tools	
Pediatric Eating Assessment Tool (PediEAT)(Thoyre et al., 2014)	2 (2)
Feeding Flock resources/questionnaires (PediEAT & others)	1 (1)
Classification systems	
EDACS (Sellers et al., 2014)	16 (16)
International Dysphagia Diet Standardisation Initiative (IDDSI) (Cichero et al., 2017)	3 (3)
Instrumental assessment tools	
Penetration Aspiration Scale (PAS) (Rosenbek et al., 1996) for VFSS	2 (2)
MBSImp (Martin-Harris et al., 2008)(for older children on VFSS)	1 (1)
Drooling tools	

Paediatric Posterior Drooling Scale (PPDS) (Koeken, 2019)	1 (1)
Drooling Quotient (van Hulst et al., 2012)	1 (1)
Outcome measures	
Therapy Outcome Measures (TOMS): Paediatric Dysphagia (Enderby and John, 2019)	1 (1)
Risk assessments	
Wolf and Glass' High Flow Nasal Cannula risk assessment	1 (1)

References:

CICHERO, J. A., LAM, P., STEELE, C. M., HANSON, B., CHEN, J., DANTAS, R. O., DUIVESTEIN, J., KAYASHITA, J., LECKO, C. & MURRAY, J. 2017. Development of international terminology and definitions for texture-modified foods and thickened fluids used in dysphagia management: the IDDSI framework. *Dysphagia,* 32**,** 293-314.

ENDERBY, P. & JOHN, A. 2019. *Therapy Outcome Measure User Guide. ,* Croydon, J& R Press Ltd.

HIBBERD, J. T., J. 2001. *Jay's Observational Assessment of Dysphagia*.

KELLY, A. 2001. *Working with Adults with Learning Disabilities*, Routledge.

KOEKEN, D. 2019. *Reliability and validity of the pediatric posterior drooling scale*.

MARTIN-HARRIS, B., BRODSKY, M. B., MICHEL, Y., CASTELL, D. O., SCHLEICHER, M., SANDIDGE, J., MAXWELL, R. & BLAIR, J. 2008. MBS measurement tool for swallow impairment—MBSImp: establishing a standard. *Dysphagia,* 23**,** 392-405.

ROSENBEK, J. C., ROBBINS, J. A., ROECKER, E. B., COYLE, J. L. & WOOD, J. L. 1996. A penetration-aspiration scale. *Dysphagia,* 11**,** 93-98.

SELLERS, D., MANDY, A., PENNINGTON, L., HANKINS, M. & MORRIS, C. 2014. Development and reliability of a system to classify the eating and drinking ability of people with cerebral palsy. *Developmental Medicine & Child Neurology,* 56**,** 245-251.

SKUSE, D., STEVENSON, J., REILLY, S. & MATHISEN, B. 1995. Schedule for oral-motor assessment (SOMA): methods of validation. *Dysphagia,* 10**,** 192-202.

THOYRE, S. M., PADOS, B. F., JINHEE, P., ESTREM, H., HODGES, E. A., MCCOMISH, C., VAN RIPER, M. & MURDOCH, K. 2014. Development and Content Validation of the Pediatric Eating Assessment Tool (Pedi-EAT). *American Journal of Speech-Language Pathology,* 23**,** 46-59.

VAN HULST, K., LINDEBOOM, R., VAN DER BURG, J. & JONGERIUS, P. 2012. Accurate assessment of drooling severity with the 5-minute drooling quotient in children with developmental disabilities. *Developmental Medicine & Child Neurology,* 54**,** 1121-1126.
