# Supplementary material for: Working With School‐Aged Children With Neurodisability and Oropharyngeal Dysphagia Who Require Mealtime Assistance: A Survey of Speech and Language Therapists’ Clinical Practice
Source: Int J Lang Commun Disord. 2026 Apr 29;61:e70254. doi: 10.1111/1460-6984.70254 (PMC13129504; doi:10.1111/1460-6984.70254)
Supplement: Supplementary file 5 — Supporting Information: jlcd70254‐supp‐0005‐SuppMat.pdf [file JLCD-61-0-s003.pdf]

Supporting Information 5: Types of carers that SLTs provide mealtime recommendations to (n=137)

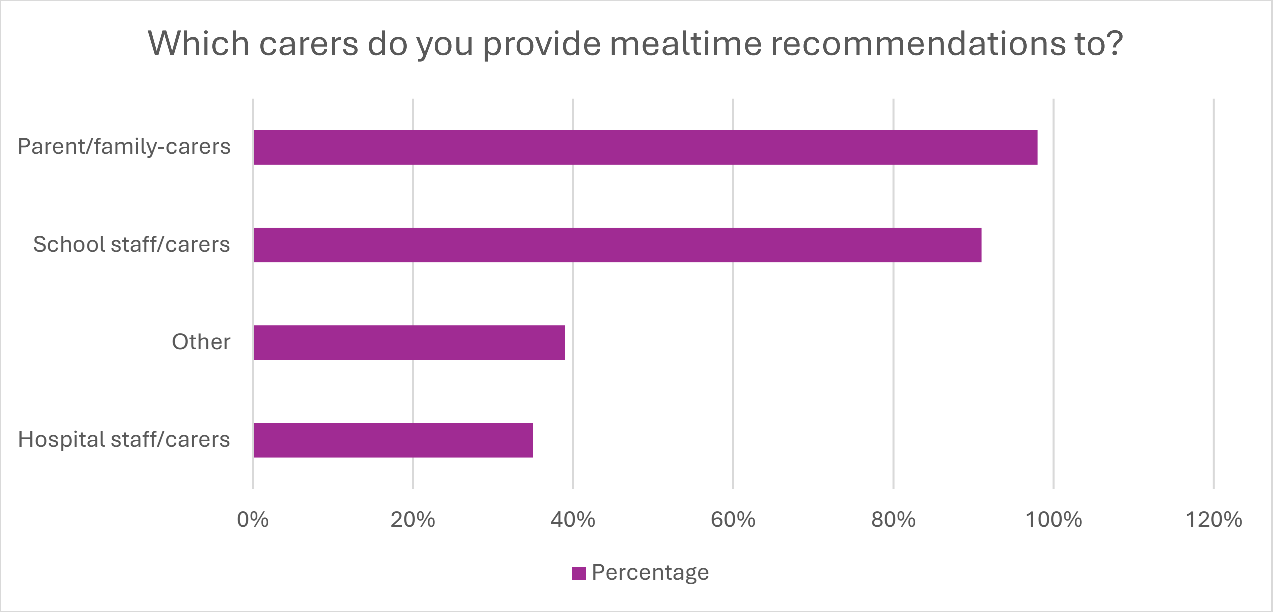

Figure: Types of carers that SLTs provide mealtime recommendations to (n=137)

Other: n = 53 (often with multiple options suggested)

Respite/short breaks/hospice: n = 35
Other home carers: n = 4
Community SLT team: n = 3
Any suitable team members: n = 4
Medical teams: n = 2
Foster carers: n = 2
Kitchen staff: n = 2
GP: n = 1
Social care: n = 1
Child: n = 1
