# Supplementary material for: Working With School‐Aged Children With Neurodisability and Oropharyngeal Dysphagia Who Require Mealtime Assistance: A Survey of Speech and Language Therapists’ Clinical Practice
Source: Int J Lang Commun Disord. 2026 Apr 29;61:e70254. doi: 10.1111/1460-6984.70254 (PMC13129504; doi:10.1111/1460-6984.70254)
Supplement: Supplementary file 6 — Supporting Information: jlcd70254‐supp‐0006‐SuppMat.pdf [file JLCD-61-0-s006.pdf]

Supporting information 6:

Interventions integrated into the meal

SOS (Toomey and Ross, 2011): n=26 (2 say adapted principles)
Talk Tools: n= 8 (4 say adapted)
Facial Oral Tract Therapy: n = 4
Oral Placement Therapy: n = 2
Bobath: n = 2
Social Stories(Gray et al., 2002): n = 2
SOFFI (Ross, 2011): n = 2
Oral hygiene: n = 1
Objects of reference: n = 1
April Winstock: n = 1
Programme of touch (oral desensitisation or stimulation): n = 1
Ark Therapeutic: n = 1
Mary Schiaveli jaw programme: n = 1
Behavioural programme: n = 1
Hanen 4 x 'S' strategy (Sussman, 2012): = 1

References:

GRAY, C., WHITE, A. L. & MCANDREW, S. 2002. *My social stories book*, Jessica Kingsley Publishers.
ROSS, E. S., & PHILBIN, M. K. 2011. Soffi: an evidence-based method for quality bottle-feedings of preterm, ill, and fragile infants. . *The Journal of perinatal & neonatal nursing,* 25**,** 349.
SUSSMAN, F. 2012. *More than words*, Toronto, Canadá The Hanen Centre.
TOOMEY, K. A. & ROSS, E. S. 2011. SOS approach to feeding. *Perspectives on Swallowing and Swallowing Disorders (Dysphagia),* 20**,** 82-87.
